# Mouthpart Ecomorphology and Predatory Behaviour in Selected Rove Beetles of the “Staphylinine Group” (Coleoptera: Staphylinidae: Staphylininae, Paederinae)

**DOI:** 10.3390/insects13080667

**Published:** 2022-07-23

**Authors:** Benedict Stocker, Sonja Barthold, Oliver Betz

**Affiliations:** Evolutionsbiologie der Invertebraten, Institut für Evolution und Ökologie, Universität Tübingen, 72076 Tübingen, Germany; benedict.stocker@student.uni-tuebingen.de (B.S.); sonja.i.d.barthold@gmail.com (S.B.)

**Keywords:** *Bisnius*, ecomechanics, ecomorphology, feeding, functional morphology, *Gyrohypnus*, leg, morphology, mouthparts, *Othius*, *Philonthus*, predation, *Quedius*, *Rugilus*, tarsus

## Abstract

**Simple Summary:**

An understanding of the evolution and diversity of organisms is vital not only in its own right, but also with regard to the way that ecosystems function and can be protected. We explore the mouthpart morphology, the feeding behaviour, and the predatory performance of several species within the hyperdiverse family of rove beetles (Staphylinidae, subfamilies Paederinae and Staphylininae) and the connections between these aspects by using scanning electron microscopy of dissected mouthparts and front legs and highspeed videography of prey-capture behaviour. Our behavioural and morphological findings indicate that the investigated representatives of the Paederinae are specialized on elusive prey such as springtails (Collembola), whereas the observed Staphylininae display characteristics more associated with generalist predation. The detected shape differences of the mandibles between the representatives of the two analysed subfamilies are correlated with predatory performance on specific types of prey. We also found correlations between body size and the preferred prey type. We describe several methods of prey capture: beetles use their front legs to attack their prey, to lift it off the ground, or to cage it. Such strategies differ among species and situations. Overall, this exploratory study provides valuable insights into the links between the morphology, behaviour, and predatory performance of rove beetles.

**Abstract:**

The representatives of the megadiverse rove beetle subfamilies Paederinae and Staphylininae (Coleoptera: Staphylinidae) are considered generalist predators, although their exact prey-capture behaviour and performance and possible links to mouthpart morphology have rarely been described. Here, we examine these relationships for selected species by SEM analyses of mouthparts and front legs and highspeed videography of prey-capture behaviour. We describe the observed behaviours and structural properties and quantify relationships between prey type, mouthpart morphology, and predatory performance based on morphometric measurements of both the shape and lever properties of the mandible. We show that the Staphylininae considered have morphological and behavioural properties generally associated with generalist predation and that the Paederinae considered display characteristics that are highly specialized on elusive prey such as Collembola. We found correlations between mandible shape and leverage, and body size and prey type. We report distinct prey-capture behaviours: the beetles use front legs and/or mandibles to attack prey, drag prey, or cage it between their legs. These strategies differ among species and situations. Overall, this exploratory study provides insights into the morphology and types of prey capture that must have played a major role in the evolution of these beetles.

## 1. Introduction

In the temperate zones and tropics, rove beetles (Staphylinidae) are among the dominant elements of the fauna of the soil, where they mainly inhabit the litter layer. With more than 63,650 described species [1], they are currently considered to be the largest insect family on earth. The megadiversity of rove beetles is exemplified by their many feeding habits, such as mycophagy, phytophagy, predation, saprophagy, algophagy, and pollenophagy [2,3,4,5]. Several major and especially species rich subfamilies (e.g., Staphylininae) have remained unexplored in terms of their comparative mouthpart morphology and (predatory) feeding behaviour. In terms of feeding behaviour and techniques, the members of the informal “Staphylinine group” are assumed to use pre-oral digestion: once the prey is captured, enzymes are discharged from the gut onto the prey while it is being masticated in the pre-oral cavity by means of a special “rotary mill” as described for some species of the genera *Megalopinus* spp. Eichelbaum, 1915 [6], *Stenus* spp. Latreille, 1797 [7] or for *Philonthus decorus* Gravenhorst, 1802 [8]. The distribution of the “rotary mill” in Staphylinidae is uncertain, although it might occur widely across the members of the “Staphylinine group”, with variations in terms of its specific mechanism and the involved morphological structures. The predatory behaviour (including the immediate prey seizure with the mandibles) of adult members of the “Staphylinine group” (sensu [5]) has seldom been observed and has only been studied in greater detail in some of its representatives among Magalopsidiinae [6], Steninae [9,10,11,12,13,14], Scydmaeninae [15,16,17], and Staphylininae [8,18,19,20]. In the present contribution, we studied the prey-capture and feeding behaviour of 20 species of beetles from different tribes of the subfamilies Staphylininae (16 species) and Paederinae (four species) and considered this behaviour in relation to their mouthpart morphology, with special emphasis on the shape of the mandibles and their bite mechanism. We did not follow a systematic taxon sampling of the examined species but instead concentrated on 55 beetles collected from their natural habitats around our study site.

In both subfamilies, generalist predation has been described except for some specialist predatory species of Staphylininae and inquiline species of Paederinae associated with feeding on the brood of their social insect hosts [5]. Generalist predatory beetles within the Staphylinidae have been observed to prey upon Nematoda, Oligochaeta, Acarina, Araneae, Collembola, Coleoptera adults, Diptera larvae, and adults and Lepidoptera larvae [5]. Similar prey can be expected to be found on the menu of beetles of the Staphylininae and Paederinae, depending on the habitats of the beetles. In rearing studies, both the larvae and adults of several species of both groups have been observed to readily accept Nematoda, Collembola, and various Diptera [21], making them attractive as research organisms that can be kept over longer periods of time for laboratory experiments. Since different prey species have different strategies of evading, escaping, and fending off a predator, a high repertoire of specializations can be expected within generalist predatory insects hunting them [22,23]. One mechanism of passive defence found among many of the above-listed prey organisms against generalist rove beetles is the development of a mechanical barrier to the mandibles of the predator, e.g., the cuticle of the head, the thorax, and the abdomen of the prey is thickened [23]. This can often be found, for example, in Acarina and adult Coleoptera. An active defence mechanism is represented by a locomotive response of the prey, i.e., it actively flees from the predator [23]. Members of the Collembola and Diptera that are possible prey of generalist staphylinids rely mostly on this type of defence. These are only two examples for the many various defence strategies of possible prey organisms [23]. Despite some specialists among the prey having evolved various and, in some cases, highly complex morphological defence structures, generalist species within the Staphylinidae are nevertheless successful when hunting such prey. A few specialists show multiple adaptations towards prey capture. For example, *Stenus* beetles have evolved a highly specific, rapidly, protrusible labium with terminal adhesive cushions to catch fast fleeing springtails [9,10,11] (reviewed in [24]). These beetles are also equipped with mandibles that are sharp and strong enough to crack the hard shells of oribatid mites [25]. These structures are similar in complexity among the various species but are often of highly differing morphology. *Philonthus marginatus* Müller, 1764 beetles, for example, use their raptorial forelimbs to catch elusive springtails. The forelimbs of the beetles are covered with thorny bristles on the inner side and have an elongated coxa and femur as well as retractable claws and adhesive tarsi [18].

By using Scanning Electron Microscopy (SEM), we aimed at providing a survey of the mouthpart morphology of selected Paederinae and Staphylininae species and evaluated their mouthparts in the context of our observations of their feeding behaviour (i.e., prey capture and food intake) towards various prey types. We hypothesize that, depending on their predominant predatory behaviour, the investigated species possess mouthparts that differ from those previously found in other (non-predaceous) subfamily groups of Staphylinidae [26]. Specific mouthpart characters such as mandible shape might function in both efficient prey capture and food processing. Moreover, since previous analyses on *Philonthus marginatus* revealed that these beetles also use their front legs for prey capture, we paid special attention to the role of these legs in other species.

Overall, this exploratory study provides valuable insights into relationships between morphology and prey capture in modern Staphylinidae and that must have played a major role in their evolutionary success.

## 2. Materials and Methods

### 2.1. Species Investigated

The beetles were collected from their natural habitats around Tübingen (Southern Germany, Baden-Württemberg) from October to December 2017 by searching through the dung of cattle and horses by hand. We also browsed sites with litter, dunghills, and garden waste with a beetle sieve.

For behavioural studies, we investigated 20 species, and for morphological studies, 15 species. In several cases, multiple specimens per species were analysed (Table 1). For morphological studies, the beetles were stored individually in Eppendorf tubes filled with 70% ethanol.

### 2.2. Morphology

#### 2.2.1. Dissection and Morphometry

The antennae were removed at the base of the scapus by using forceps. The heads of the larger specimens were boiled in 10% potassium hydroxide solution for 1–3 min. Subsequently, they were rinsed in distilled water. The head, labrum, labium, maxillae, and mandibles were dissected using fine needles or sharpened tungsten filaments, depending on the size of the specimen. Only the left front legs were separated at the joint between the femur and tibia by using forceps, other legs were not considered. More details concerning the microdissection methods can be found in [27]. Only the left side of each beetle was dissected. The mandibles of all dissected specimens were analysed, whereas for all the other parts (leg, labrum, maxilla, and labium), only one in good condition was chosen and prepared for further analysis.

**Figure 1:**
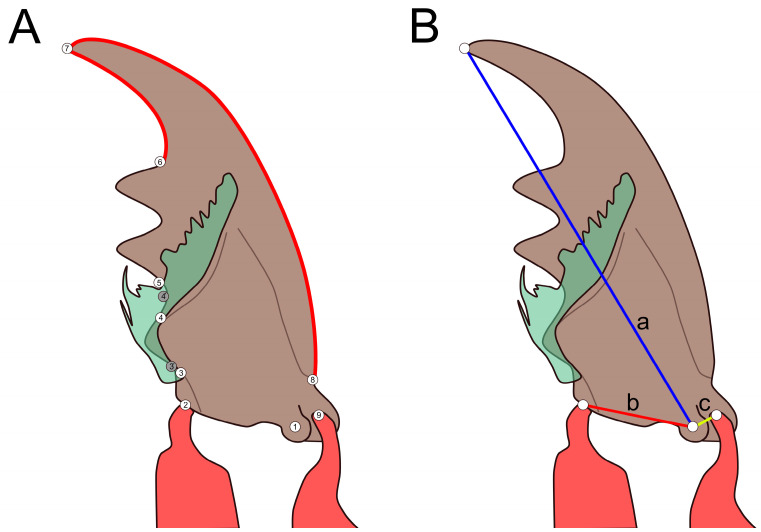
(**A**) Scheme of a measured mandible with registered landmarks (numbers). Ventral aspect of left mandible. Red lines with semilandmarks, 3′ as alternative landmark 3, 4′ as alternative landmark 4 in cases where a brush-like prostheca was not present (cf. *Rugilus* spp.), (**B**) Scheme of registered landmarks with levers, ventral view of left mandible, a = mandible length, blue; b = in-lever, red; c = out-lever, yellow.

In addition, length measurements of the pronotum and the whole body, the forebody (length between anterior end of head to posterior end of elytra along the sagittal plane), the elytra, and of the head length and head width were taken using a stereomicroscope (Leica Microsystems MZ125, Wetzlar, Germany) with an integrated, calibrated microscale. Measurements of mandible width (between landmarks 2 and 9, Figure 1A) and incisor length were taken from SEM images by means of Fiji (version 1.0, [28]). Ratios between measurements were created to eliminate size effects (Appendix A, Table A1). The specimens are stored in 70% ethanol in our institute in Tübingen, Germany.

#### 2.2.2. Scanning Electron Microscopy (SEM) and Sample Preparation for Descriptions and Measurements

The dissected parts were dried in an ascending ethanol series (70%, 80%, 90%, and 100% (twice)) followed by critical point drying in CO_2_ (Polaron Critical Point Dryer, Quorum Technologies, Laughton, UK). They were then cleaned by being submerged in 30% hydrogen peroxide solution for 5 min. The objects were dehydrated again for 5 min in 100% ethanol.

The specimens were mounted on a roof-like structure of silver tape attached to a standard SEM object mount. They were stabilized using conductive silver paint (all materials from Plano GmbH, Wetzlar, Germany). Objects were gold-coated for 5 min from various angles to improve electrical properties (Quorum Emitech K550X, Ashford, UK).

Images were taken using a Scanning Electron Microscope (ZEISS EVO LS 10, Oberkochen, Germany) by using the secondary electron detector and, if necessary, the backscatter detector. An acceleration voltage of 15 kV was used.

The terminology and method for mouthpart descriptions follows those of [26].

#### 2.2.3. Landmark Measurements

Landmarks (Figure 1A) were digitized using tpsDig2 (Version 2.31, [29]). Semilandmarks were placed between landmarks six and seven (15 semilandmarks) and between landmarks seven and eight (30 semilandmarks), the number of landmarks was chosen to provide a good representation of the actual shape of the mandible. Since the examined *Rugilus* species do not possess a brush-like prostheca, we set alternative landmarks 3′ and 4′ that delimited the third tooth of the retinaculum located in the region in which the other species had a prostheca (Figure 1A). The mandible shapes were compared using tpsRelw (Version 1.70, [30]). A consensus was generated and differences in shape were visualized. Differences were quantified using the bending energy method. A principal component analysis (PCA) was performed in tpsRelw. The two principal components (referred to as PC1 and PC2) explaining most of the variation were selected for a 2D representation of the shape space. Semilandmarks were not weighted equally to regular landmarks but defined as sliders while generating a consensus. To examine differences between (sub-)tribes, a Mahalanobis distance was calculated using a canonical variate analysis (CVA) with 1,000,000 permutations in MorphoJ (1.07a, [31]).

#### 2.2.4. Lever Calculations

Lever arm lengths were measured using distances between landmarks. Three levers were measured, i.e., mandible length (a), in-lever (b), and out-lever (c) (Figure 1B). The in-lever length is the distance between the ventral condyle and the attachment point of the adductor muscle, the out-lever length is the distance between the ventral condyle and the attachment point of the abductor muscle, whereas the mandible length is the distance between the ventral condyle and the mandibular apex (Figure 1B). Ratios between the in- and out-levers and between the levers and the mandible length were calculated to eliminate size differences. Measurements were taken using tpsDig2 (Version 2.31, [29]) and Fiji (version 1.0, [28]). This approach was an attempt to apply the lever model proposed by [32] for two-dimensional SEM images.

#### 2.2.5. Statistical Comparisons

**Figure 2:**
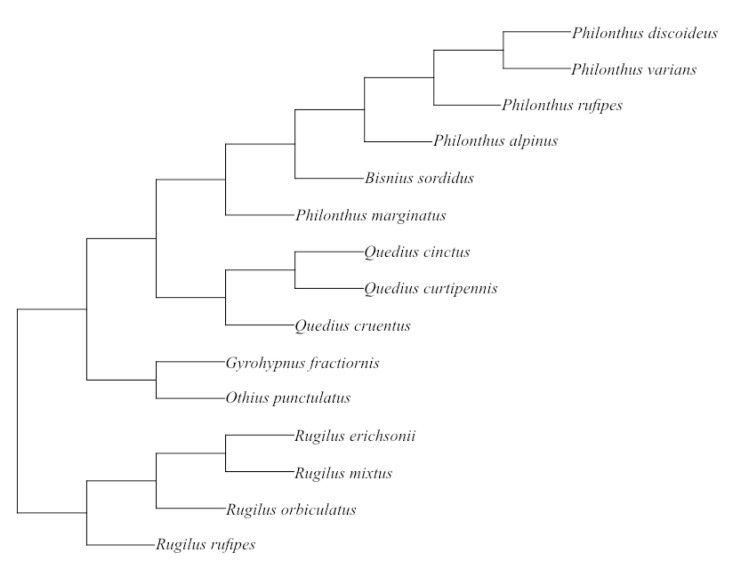
Phylogenetic scheme for the considered species reconstructed from various literature sources as cited in the text.

For species with multiple individuals, species data are represented as mean values with the standard deviation (Appendix A, Table A1). The data are body-size corrected, as only ratios were used for analysis. As data for species cannot be treated as independent [33,34], we analysed our results by using phylogenetic comparative methods. With this aim, we compiled a phylogenetic scheme of the investigated species from various literature sources (Figure 2). We followed [35] and [36] as well as [37] to establish the relationships within and between the genera *Quedius, Othius, Gyrohypnus,* and *Bisnius* and the external relationships between the aforementioned genera and *Philonthus* and *Rugilus*. The phylogenetic relationships of the species within *Philonthus* and *Rugilus* were approximated using taxonomic information [38]. For the analysis, branch lengths values were set to one, because they could not be calculated for our phylogenetic scheme [39]. Phylogenetic independent contrasts (PICs) were calculated using the “ape” package (version 5.0, [40]) in RStudio (Version 1.2.5001, [41]). Since the data, even after logarithmic and arcsine transformation, did not fulfil the required normal distribution for parametric PGLS methods, Spearman rank-sum correlations were calculated by means of the “Hmisc” package (version 4.4-1, [42]) in R. A stepwise discriminant function analysis (DA) was performed on the log10-transformed morphometric ratios (Appendix A, Table A1) and on the scores of the geometric morphometric PCA (PC1, PC2) in order to analyse the predictive properties for tribe membership of the morphometrical measurements by using SPSS 27 (version 27.0.0.0, [43]).

Plots were made using the “ggplot2” (version 3.3.3, [44]) package in R with the original non-transformed data.

### 2.3. Prey-Capture Behaviour

#### Observations and Highspeed Recordings

**Figure 3:**
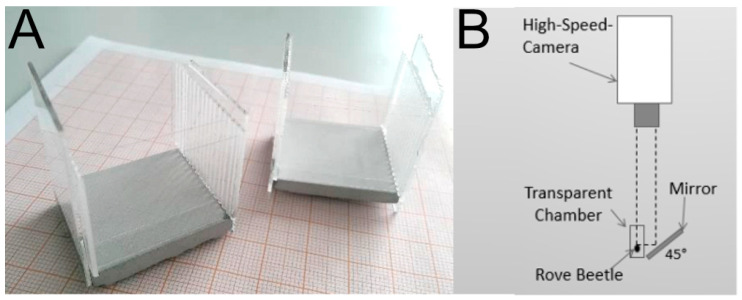
(**A**) Chambers used for filming were adjustable in width to accommodate the various sizes of beetles. They were all 35 mm in length and 25 mm in height. A microscopy cover glass was used to close the top of the chamber to prevent beetles from escaping. (**B**) Experimental setup for the filming process (side view).

The events of beetles capturing prey were filmed with a highspeed camera (Photron FastCam SA3120K-K2; measurements on 500 fps, Pfullingen, Germany) equipped with a macro lens. During the filming, the beetles were kept in small width-adjustable chambers (2.5 cm height and 3.5 cm length) that were fabricated of gypsum and microscopy glassware (Figure 3A). Inside the chambers, the beetles were filmed with the camera attached above. A mirror was placed at an angle of 45° nearby to provide recordings of the scene from various perspectives (Figure 3B) (cf. [45]). Infrared light at 850 nm (medium range), which is outside of the visible light spectrum for most insects [46], provided by a security lamp (Bosch IR Illuminator 5000 MR; Grasbrunn, Germany) was used for illumination during the filming in order not to distract the animals with unnatural light conditions.

The diverse range of types of potential prey found in the natural habitats of the beetles and possible specifications of the beetles were considered by offering them three different prey species. The species used as prey were (1) slowly moving and soft-skinned larvae of *Drosophila melanogaster* Meigen, 1830 (Diptera: Drosophilidae), (2) slowly moving and hard-shelled mites of *Archegozetes longisetosus* Aoki, 1865 (Oribatida: Trhypochthoniidae) and (3) soft-skinned elusive springtails of *Heteromurus nitidus* Templeton, 1835 (Collembola: Entomobryidae). Prior to the experiments and before being confronted with the beetles, the mites were treated with hexane for 60 s to disable their natural defence mechanism of spraying defensive secretions at predators, thereby leaving their hard shell as their only defence (cf. [25]).

In each prey-capture trial, the beetles were left together with at least one individual of one of the three prey in the transparent chambers for at least 10 min. This was repeated three times for each prey type per beetle to enhance the chance of observing the beetle attacking the prey. We therefore achieved a total of nine trials per beetle evenly distributed across the three prey types. Individuals that showed only low activity during the main experiments or were not recorded in sufficient quality were provided another 1–3 opportunities to perform towards the potential prey. Between the repeats and before the first day of observation, all individuals were kept isolated under starvation conditions for three days before the recordings to increase their feeding motivation. Following the observational experiments, the beetles were killed by being placed in a refrigerator and were transferred to 70% ethanol for later species identification [38]. Definitions and descriptions of the observed behaviours were derived by subsequent careful observation of the videos.

The percentage of prey capture success per prey type was calculated by dividing the number of each prey type successfully captured by the total number of prey animals captured per species, each individual was offered all types of prey three times for 10 min.

## 3. Results

### 3.1. Comparative Mouthpart and Leg Morphology

In the following we provide SEM images and descriptions of the front leg (Figure 4 and Figure 5), labrum (Figure 6 and Figure 7), mandible (Figure 8 and Figure 9), maxilla (Figure 10 and Figure 11) and the labium-hypopharynx (Figure 12 and Figure 13) of the dissected specimens. 

#### 3.1.1. Bisnius Sordidus

**Figure 4:**
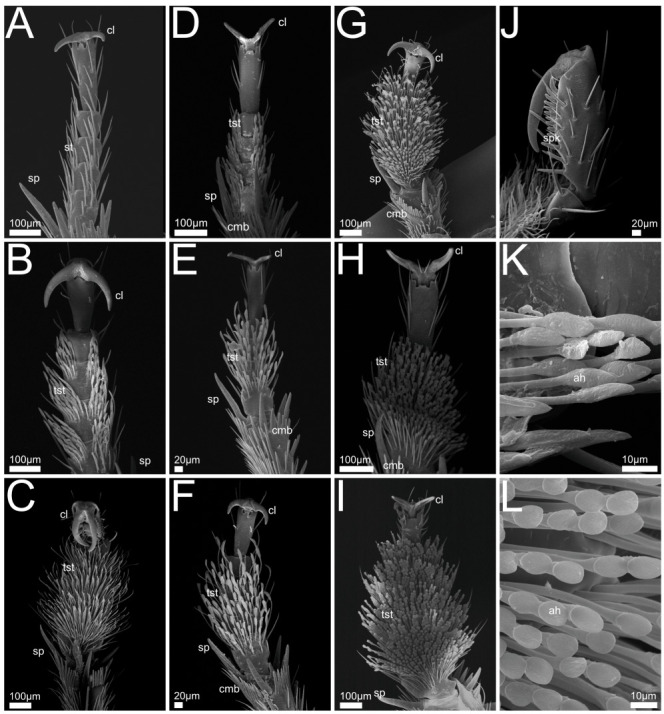
SEM views of Staphylinini front legs. Images **A**–**I**, ventral view of claw, tarsomeres and end of tibia (left front leg), **J**–**L** with detailed views of adhesive setae and claw, (**A**) *Bisnius sordidus* (female); (**B**) *Philonthus varians* (female); (**C**) *P. marginatus* (male); (**D**) *P. rufipes* (female); (**E**) *P. discoideus* (male), (**F**) *P. alpinus* (male); (**G**) *Quedius cinctus* (male); (**H**) *Q. cruentus* (female); (**I**) *Q. curtipennis* (female); (**J**) *P. marginatus* (male), lateral view of claw (left front leg); (**K**) *P. varians* (female), adhesive setae on ventral tarsomere surface (left front leg); (**L**) *Q. cruentus* (female), adhesive setae on ventral tarsomere surface (left front leg). Abbreviations: ah: adhesive spatulate head; tst: tenent setae; cl: claw; cmb: comb-like structure; sp: spur; spk: spikes; st: setae (non-adhesive); tst: tenent setae.

Front leg (Figure 4A): Claw symmetrical, curving ventro-mediad, paired long setae extending laterad on the dorsal side. Leg with five tarsomeres, 1–4 laterally with groups of long setae, apical tarsomere elongated and with lateral long setae, end of tibia with long spur and several shorter spurs directed distad.

Labrum (Figure 6A): Free, connected to clypeus by clypeolabral suture. Anterior margin rounded with medial emargination, anteriorly with long setae directed distad and curved mediad, length of hairs decreasing mediad. Epipharynx covered with hair-like trichomes directed mediad and distad, lateral and anterior margins smooth.

Mandible (Figure 8A): Mandibular apex acute, oriented mesad. Cutting edge (incisor area) and retinaculum present, subapical tooth absent. Retinaculum tooth-like. Lobe-like prostheca developed as a brush with hair-like trichomes oriented distad. Mola absent.

Maxilla (Figure 10A,J): Cardo transverse. Stipes subdivided into basi- and mediostipes, the latter forming the base of both galea and lacinia. Lacinia fused with mediostipes, reaching about half the length of the galea. Galea at distal margin of mediostipes, reaching second palpomere. Galea apically differentiated as a robust brush with mediad curved setae. Lacinia apico-mesally differentiated as robust brush with mediad curved setae, reaching medial area of galea. Palpifer slender and medially fused with basi- and mediostipes. Maxillary palp with four palpomeres, basal palpomere small, apical palpomere with short sensilla and apical receptor bundle with campaniform receptors and trichoid sensilla.

Labium-hypopharynx (Figure 12A,J): Prementum with longitudinal dorsal fold, medial rows of hairs reaching up to anterior margin. Lateral margin with two distinct parallel rows of mesad- and dorsad-directed spines and trichomes.

Palps directed distad, with three palpomeres. Second palpomere with mediad-directed setae, apical palpomere with campaniform receptors and trichoid sensilla, and apical receptor bundle with papilliform receptors and sensilla. Ligula of prementum in between antero-lateral lobes as medial unpaired projection equipped with papilliform receptors.

Separation towards hypopharynx by transverse suture. Anterior hypopharynx with smooth medial surface, laterally delimited by mediad-directed trichomes.

Hypopharynx proximally delimited by anteriorly directed hair-like trichomes, late-rally delimited by mediad-directed trichomes.

#### 3.1.2. *Philonthus* spp.

Front leg (Figure 4B–F,J,K): Claw symmetrical, curving ventro-mediad, or with claw curving proximad and apical tarsomere with two paired lateral rows of spikes, each side of the claw fitting between two of the rows (*P. marginatus*, Figure 4C,J). Five tarsomeres, 1–4 medially with tenent setae, laterally with few long unmodified setae or medial margin smooth and tenent setae only appearing laterally (*P. varians*, Figure 4B, *P. rufipes*, Figure 4D), tarsomere five without tenent setae, smooth and with lateral long setae or with spikes (*P. marginatus*, Figure 4C,J). End of tibia with long and several shorter spurs and comb-like structures directed distad.

Labrum (Figure 6B–F,J,K): Free, connected to clypeus by clypeolabral suture. Shape rectangular (*P. alpinus*, Figure 6F,K), anterior margin protruding laterad (*P. varians*, Figure 6B), medially slightly or deeply (*P. marginatus*, Figure 6C) emarginate. Apically with several long setae, oriented distad, curving mediad, length decreasing mediad. Anterior epipharynx covered with mesad and anteriorly directed trichomes, lateral anterior margin smooth.

Mandible (Figure 8B–F,J): Slender, crescent-shaped (*P. marginatus*, Figure 8C), robust (*P. discoideus*, Figure 8E), or with wide base up to retinaculum and tapered towards apex. Mandibular apex acute and oriented mediad, cutting edge (incisor area) connecting apex with retinaculum, subapical tooth absent.

Retinaculum developed as two or one (*P. alpinus*, Figure 8F) mostly pronounced tooth-like protrusions. Lobe-like prostheca oriented mesad and distad, developed as a flexible brush with hair-like trichomes. Mola absent.

Maxilla (Figure 10B–F): Cardo transverse. Stipes subdivided into basi- and mediostipes, the latter forming the base of both galea and lacinia. Galea apically differentiated as a robust brush with curved setae, reaching second palpomere of maxillary palp. Lacinia extending up to approximately half the length of galea or only basal area (*P. marginatus*, Figure 10C, *P. alpinus*, Figure 10F), apico-mesally differentiated as a robust brush with curved setae. Palpifer slender and medially fused with basi- and mediostipes.

Maxillary palp with four palpomeres, basal palpomere short, first three palpomeres with long setae, apical palpomere with papilliform receptors, apically with receptor bundle embedded in soft pad (similar to *Bisnius* as shown in Figure 10J).

Labium-hypopharynx (Figure 12B–F,K,L): Prementum with longitudinal dorsal suture with medial parallel rows of setae, extending up to anterior margin. Lateral margin with many mesad- and dorsad-directed spines and trichomes.

Palps directed distad, three palpomeres. Second palpomere with mediad-directed setae, apical palpomeres with papilliform receptors and sensilla, and apical receptor bundle with papilliform receptors and sensilla. Ligula of prementum in between antero-lateral lobes as medial unpaired projection equipped with papilliform receptors.

Separation towards hypopharynx by transverse suture. Anterior hypopharynx with smooth medial surface, delimited laterally by mediad-directed trichomes. Hypopharynx delimited laterally and proximally by anteriorly directed hair-like trichomes.

#### 3.1.3. *Quedius* spp.

Front leg (Figure 4G–I,L): Claw symmetrical, curving ventro-mediad, or mediad (*Q. cruentus*, Figure 4H), with blunt ends (*Q. cruentus*, Figure 4H) or acute, with paired long setae extending laterad from empodium. Five tarsomeres, 1–4 medially and up to lateral margin with tenent setae with discoid tips, laterally with few long unmodified setae. Tarsomere five without tenent setae. End of tibia with long and several shorter spurs and comb-like structures directed distad.

Labrum (Figure 6G–I,L): Free, connected to clypeus by clypeolabral suture. Shape, rectangular (*Q. curtipennis*, Figure 6I), anterior margin protruding laterad (*Q. cinctus*, Figure 6G, *Q. cruentus*, Figure 6H), medially with slight emargination (*Q. cinctus*, Figure 6G). Apically with several long setae, oriented distad, curving mediad, length decreasing mediad. Anterior epipharynx covered with mesad and anteriorly directed trichomes, lateral anterior margin smooth (*Q. curtipennis*, Figure 6I) or covered with trichomes.

Mandible (Figure 8G–I,K): Long, crescent-shaped (*Q. curtipennis*, Figure 8I) or slender (*Q. cinctus*, Figure 8G) or with wide basal and medial area and a tapered shape towards the apex. Mandibular apex acute and oriented mediad, cutting edge (incisor area) connecting apex with retinaculum, subapical tooth absent.

Retinaculum developed as two pronounced tooth-like projections. Lobe-like prostheca oriented mesad and distad or laterad (*Q. cruentus*, Figure 8H), developed as a flexible brush with hair-like trichomes. Mola absent.

Maxilla (Figure 10G–I,K): Cardo transverse. Stipes subdivided into basi- and mediostipes, the latter forming the base of both galea and lacinia. Galea apically differentiated as a robust brush with curved setae, reaching third palpomere of maxillary palp. Lacinia apico-mesally differentiated as a robust brush with curved setae, extending up to approximately half the length of galea. Palpifer slender, medially fused with basi- and mediostipes. Maxillary palp with four palpomeres, basal palpomere short, first three palpomeres with long setae, apical palpomere with long slit-like receptors (*Q. cinctus*, Figure 10G,K) and papilliform receptors, apically with receptor bundle of sensilla embedded in soft pad.

Labium-hypopharynx (Figure 12G–I): Prementum with medial papilliform receptors or missing in *Q. curtipennis* (Figure 12I), suture along anterior margin (*Q. cinctus*, Figure 12G). Lateral margin with many mesad- and dorsad- (on the outside laterad)directed spines and hair-like trichomes, converging proximally (*Q. cinctus*, Figure 12G), sometimes arranged in two rows (*Q. cruentus*, Figure 12H, *Q. curtipennis*, Figure 12I). Palps directed distad, three palpomeres. Second palpomere with distad-directed setae, apical palpomere elongated with campaniform sensilla and apical receptor bundle with papilliform receptors and trichoid sensilla. Ligula of prementum in between antero-lateral lobes as medial unpaired projection equipped with papilliform receptors. Separation towards hypopharynx by transverse suture and transverse row of spines (*Q. cinctus*, Figure 12G). Anterior area of hypopharynx with proximad- and mesad-directed hair-like trichomes (*Q. cruentus*, Figure 12H) or smooth with lateral rows of mesad-directed hair-like trichomes. Proximal area of hypopharynx with mesad-directed and anteriorly directed hair-like trichomes (*Q. cruentus*, Figure 12H) or smooth with lateral rows of mesad-directed hair-like trichomes. Hypopharynx proximally delimited by transverse row of anteriorly directed hair-like trichomes.

#### 3.1.4. *Gyrohypnus fracticornis*

**Figure 5:**
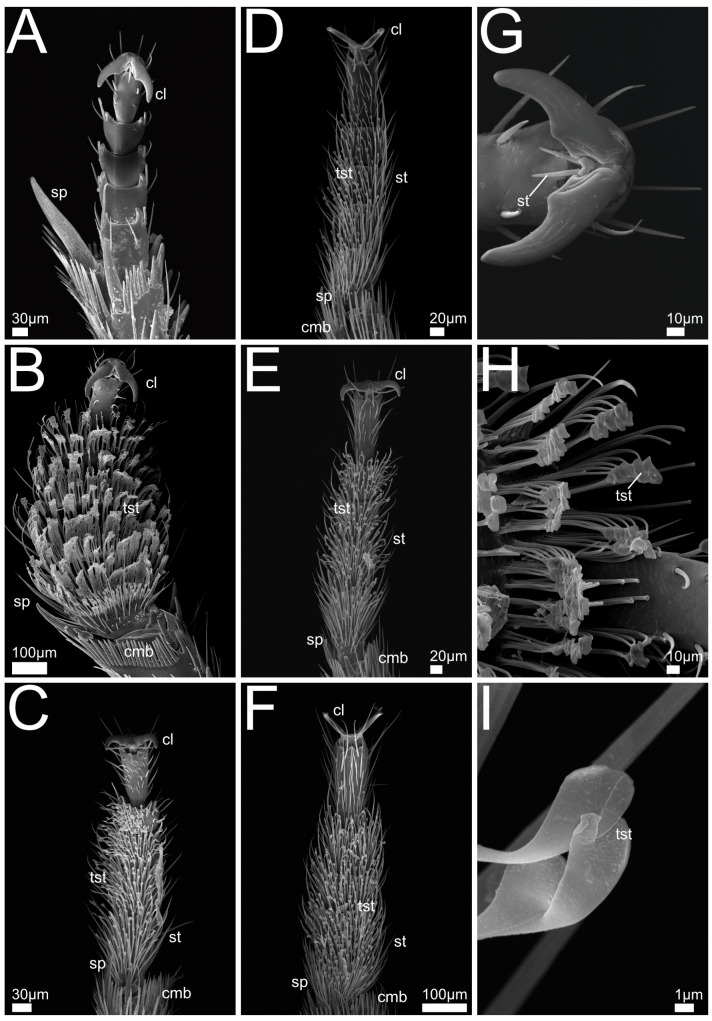
SEM views of Xantholinini, Othiini and Stilicina front legs. Images **A**–**F** with ventral view of claw, tarsomeres and end of tibia (left front leg). **G**–**I**, detailed views of adhesive setae and claw, (**A**) *Gyrohypnus fracticornis* (male); (**B**) *Othius punctulatus* (male); (**C**) *Rugilus erichsonii* (male); (**D**) *R. mixtus* (male); (**E**) *R. orbiculatus* (male); (**F**) *R. rufipes* (male); (**G**) *G. fracticornis* (male), ventral view of claw (left front leg); (**H**) *O. punctulatus* (male), ventral view of fourth and fifth tarsomere; (**I**): *R. orbiculatus* (male), ventral view of adhesive spatulate seta heads. Abbreviations: cl: claw; cmb: comb-like structure; sp: spur; st: setae (non-adhesive); tst: tenent setae.

**Figure 6:**
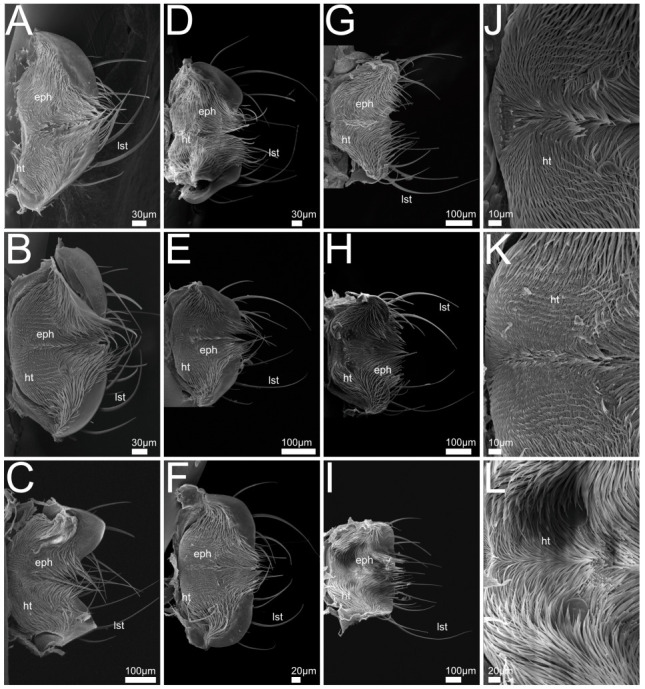
SEM views of Staphylinini labrum. Images **A**–**I** epipharynx, **J**–**L** with detailed views of medial area. (**A**) *Bisnius sordidus*; (**B**) *Philonthus varians*; (**C**) *P. marginatus*; (**D**) *P. rufipes*; (**E**) *P. discoideus*, (**F**) *P. alpinus*; (**G**) *Quedius cinctus*; (**H**) *Q. cruentus*; (**I**) *Q. curtipennis*; (**J**) *P. discoideus*; (**K**) *P. alpinus*; (**L**) *Q. curtipennis*. Abbreviations: eph: epipharynx; ht: hair-like trichomes; lst: long setae.

Front leg (Figure 5A,G): Claws symmetrical, curving ventro-mediad with paired long setae extending laterad from empodium. Five tarsomeres, tarsomere 1 with medial setae, tarsomeres 2–4 laterally with paired long setae, a spur, and medial setae on basal tarsomere. End of tibia with long spur and several smaller spurs and comb-like structures directed distad.

**Figure 7:**
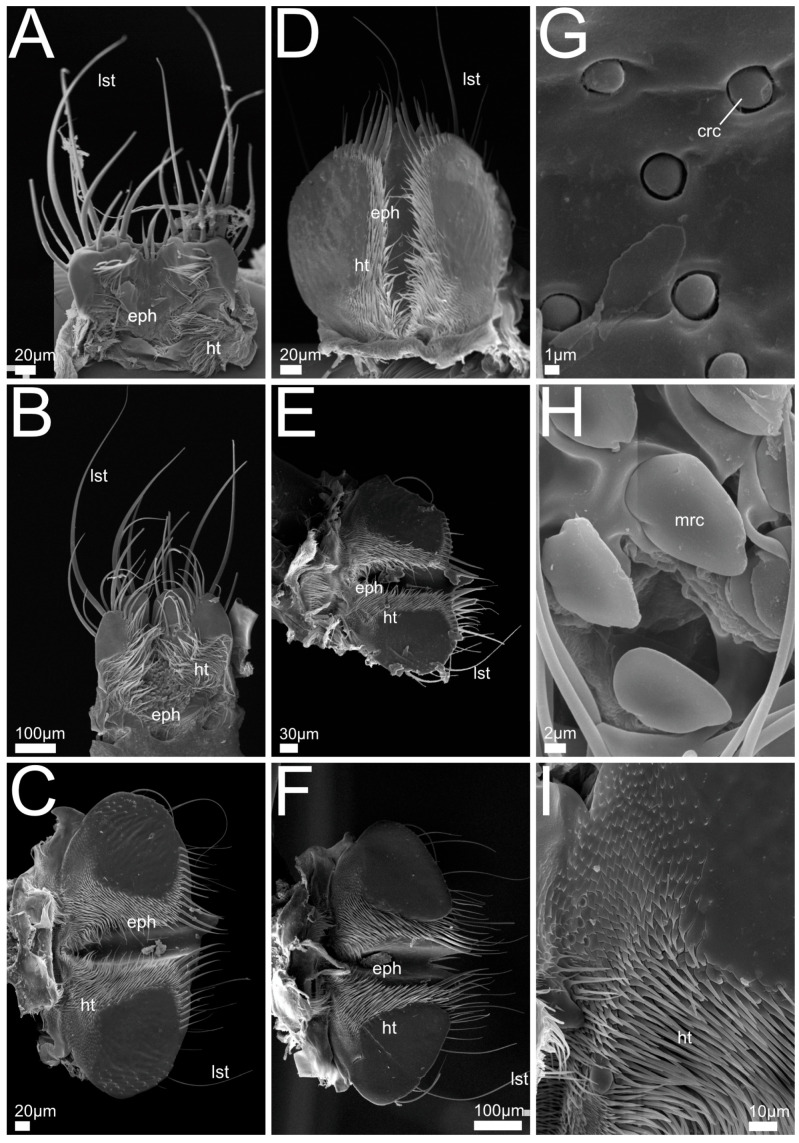
SEM views of Xantholinini, Othiini and Stilicina labrum. Images **A**–**F** epipharynx, **G**–**I** with detailed views of trichomes and receptors. (**A**) *Gyrohypnus fracticornis*; (**B**) *Othius punctulatus*; (**C**) *Rugilus erichsonii*; (**D**) *R. mixtus*; (**E**) *R. orbiculatus*; (**F**) *R. rufipes*; (**G**) *G. fracticornis*, medial aspect of epipharynx; (**H**) *O. punctulatus*, medial aspect of epipharynx; (**I**) *R. erichsonii*, medio-lateral aspect of epipharynx. Abbreviations: crc: campaniform receptors; eph: epipharynx; ht: hair-like trichomes: lst: long setae; mrc: mechanoreceptors.

**Figure 8:**
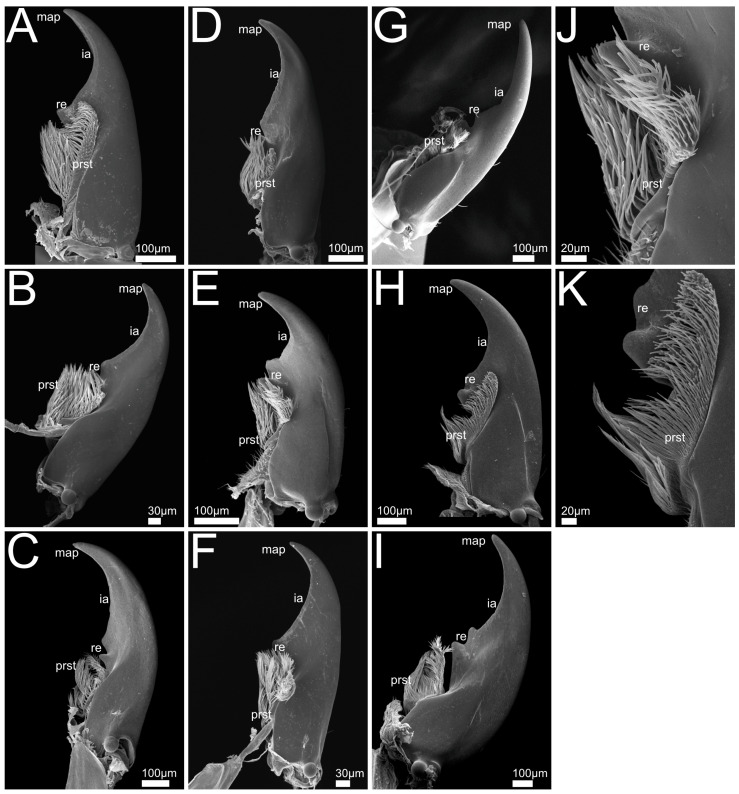
SEM views of Staphylinini mandible. Images **A**–**I** ventral aspect of mandible, **J**–**K** with detailed views of prostheca. (**A**) *Bisnius sordidus*; (**B)** *Philonthus varians*; (**C**) *P. marginatus*; (**D**) *P. rufipes*; (**E**) *P. discoideus*, (**F**) *P. alpinus*; (**G**) *Quedius cinctus*; (**H**) *Q. cruentus*; (**I**) *Q. curtipennis*; (**J**) *P. discoideus*, ventral aspect of prostheca; (**K**) *Q. cruentus*, ventral aspect of prostheca. Abbreviations: ia: incisor area; map: mandibular apex; prst: prostheca; re: retinaculum.

Labrum (Figure 7A,G): Free, connected to clypeus by clypeolabral suture. Anterior margin rectangular with rounded corners and medial emargination. Apically with several long setae protruding distad and curving mediad and a pair of shorter medial setae originating from the emargination.

Anterior epipharynx with lateral areas covered with hair-like setae oriented mesad, medially with field of campaniform receptors. Proximal epipharynx laterally with folds oriented laterad and distad, epipharynx concluded proximally by a fold with distad-directed trichomes.

**Figure 9:**
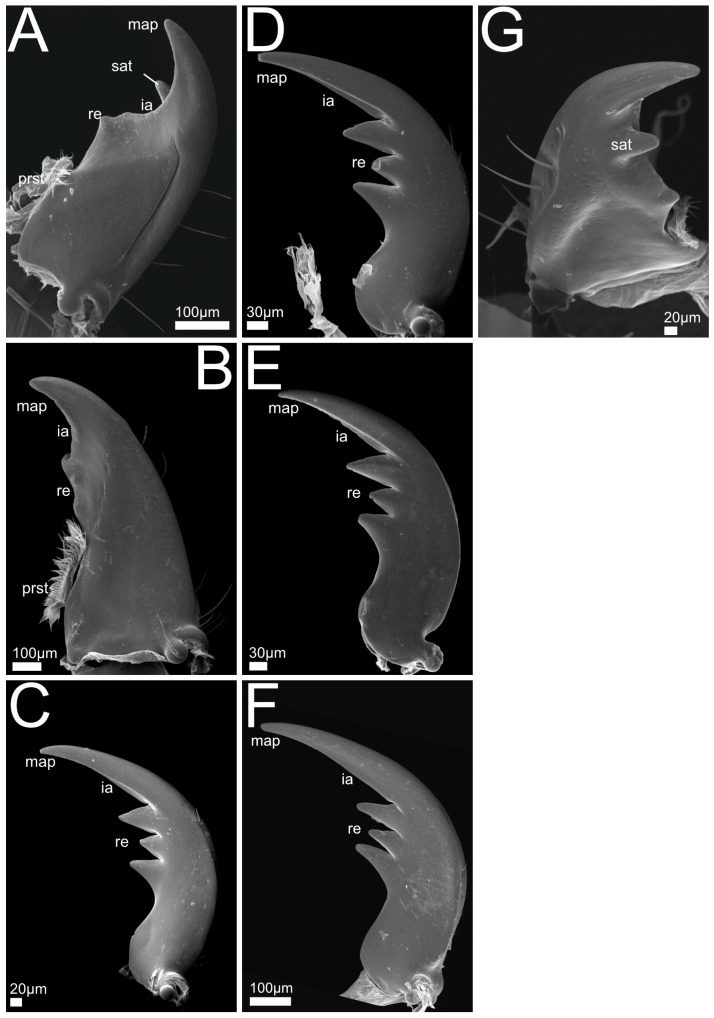
SEM views of Xantholinini, Othiini and Stilicina mandible. Images **A**–**F** ventral, (**G**) dorsal aspects of mandible. (**A**) *Gyrohypnus fracticornis*; (**B**) *Othius punctulatus*; (**C**) *Rugilus erichsonii*; (**D**) *R. mixtus*; (**E**) *R. orbiculatus*; (**F**) *R. rufipes*; (**G**) *G. fracticornis*. Abbreviations: ia: incisor area; map: mandibular apex; prst: prostheca; re: retinaculum; sat: subapical tooth.

**Figure 10:**
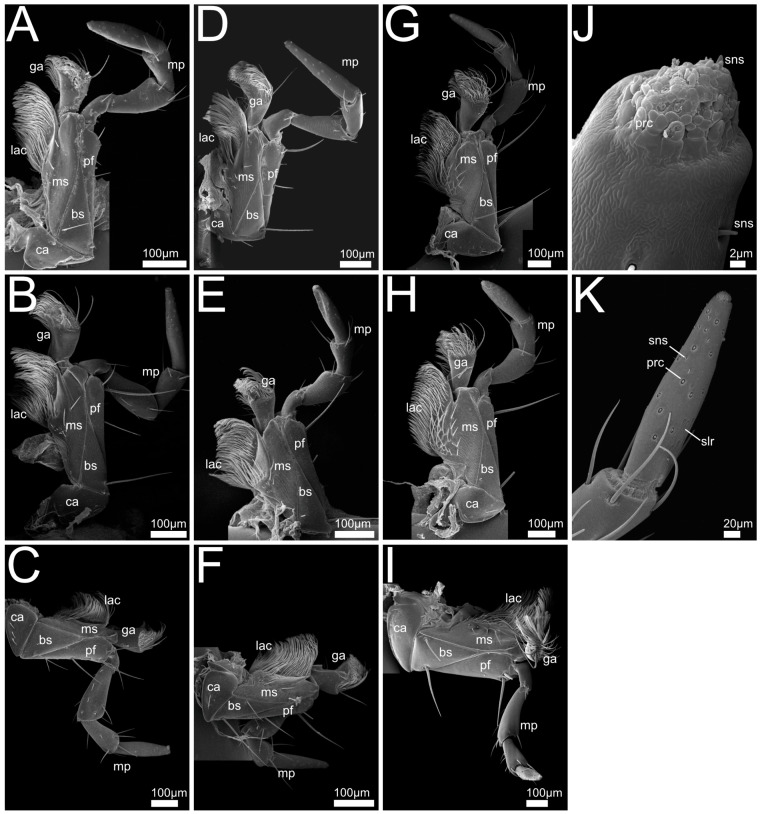
SEM views of Staphylinini maxilla. Images **A**–**I** ventral aspect of maxilla **J**–**K** with detailed views of apical palpomeres and receptors. (**A**) *Bisnius sordidus*; (**B**) *Philonthus varians*; (**C**) *P. marginatus*; (**D**) *P. rufipes*; (**E**) *P. discoideus*, (**F**) *P. alpinus*; (**G**) *Quedius cinctus*; (**H**) *Q. cruentus*; (**I**) *Q. curtipennis*; (**J**) *B. sordidus*, apical receptor bundle; (**K**) *Q. cinctus*, apical segment of maxillary palp. Abbreviations: bs: basistipes; ca: cardo; ga: galea; lac: lacinia; ms: mediostipes; mp: maxillary palp; pf: palpifer; prc: papilliform receptor; slr: slit-like receptors; sns: sensilla.

Mandible (Figure 9A,G): Slightly asymmetrical, long. Mandibular apex acute and oriented mesad. Incisor area acute, connecting apex with retinaculum. Subapical tooth present as acute projection that dorsally adjoins incisor area (Figure 9G). Retinaculum developed as two tooth-like projections proximal of incisor area. Lobe-like prostheca developed as a flexible brush with hair-like trichomes. Mola absent.

**Figure 11:**
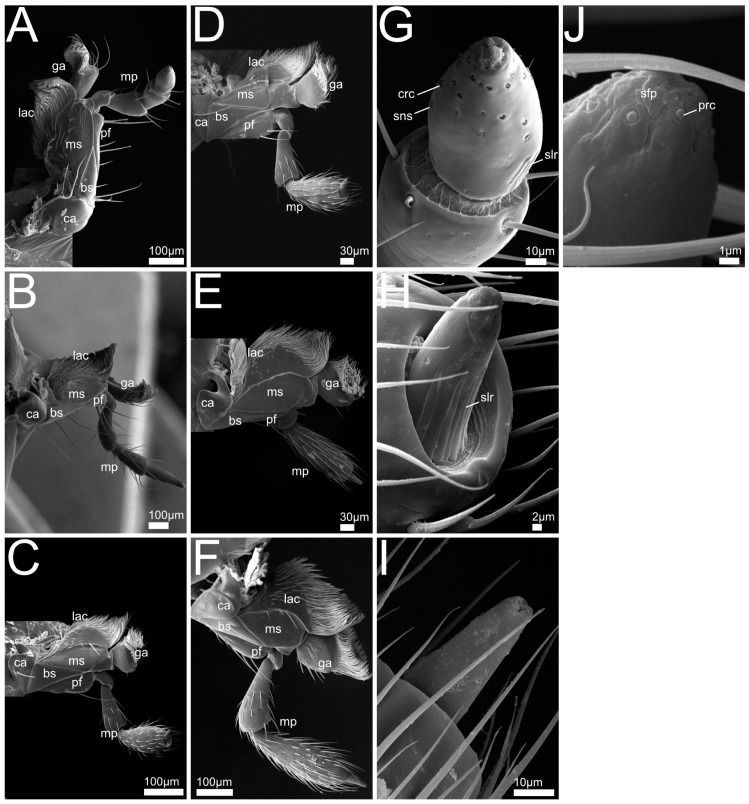
SEM views of Xantholinini, Othiini and Stilicina maxilla. Images **A**–**F** ventral aspect of maxilla, **G–J** with detailed views of apical palpomeres and receptors. (**A**) *Gyrohypnus fracticornis*; (**B**) *Othius punctulatus*; (**C**) *Rugilus erichsonii*; (**D**) *R. mixtus*; (**E**) *R. orbiculatus*; (**F**) *R. rufipes*; (**G**) *G. fracticornis*, apical segment of maxillary palp; (**H**) *R. erichsonii*, apical segment of maxillary palp; (**I**) *R. orbiculatus*, apical segment of maxillary palp; (**J**) *R. mixtus*, apical receptor bundle of maxillary palp. Abbreviations: bs: basistipes; ca: cardo; crc: campaniform receptor; ga: galea; lac: lacinia; slr: slit-like receptors; ms: mediostipes; mp: maxillary palp; pf: palpifer; prc: papilliform receptor; sfp: soft pad; sns: sensilla.

**Figure 12:**
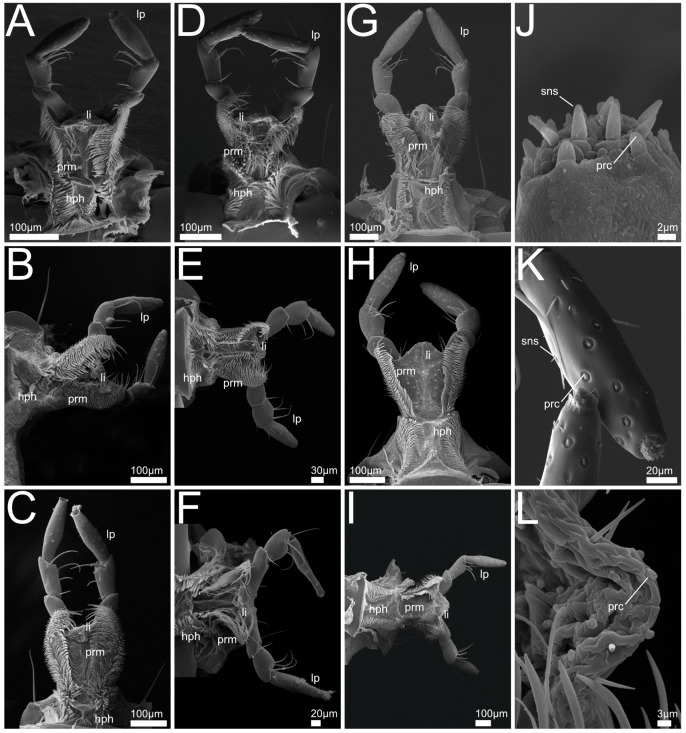
SEM views of Staphylinini labium-hypopharynx. Images **A**–**I** dorsal aspect of labium-hypopharynx, **J–L** with detailed views of receptors on apical palpomeres and ligula, (**A**) *Bisnius sordidus*; (**B**) *Philonthus varians*; (**C**) *P. marginatus*; (**D**) *P. rufipes*; (**E**) *P. discoideus*; (**F**) *P. alpinus*; (**G**) *Quedius cinctus*; (**H**) *Q. cruentus*; (**I**) *Q. curtipennis*; (**J**) *B. sordidus*, apical receptor bundle; (**K**) *P. rufipes*, apical segments of labial palpi; (**L**) *P. discoideus*, dorsal aspect of anterior prementum and ligula. Abbreviations: hph: hypopharynx; li: ligula; lp: labial palp; prc: papilliform receptor; prm: prementum; sns: sensilla.

Maxilla (Figure 11A,G): Cardo transverse. Stipes subdivided into basi- and mediostipes, the latter forming the base of both galea and lacinia. Lacinia apico-mesally differentiated as a robust brush with mediad- and, in the basal region, distad-oriented curving setae. Lacinia extending slightly beyond base of galea.

Galea at distal margin of mediostipes, extending up to approximately half the length of the maxillary palp. Galea apically differentiated as a robust brush with mediad-curving setae. Palpifer slender and medially fused with basi- and mediostipes.

Maxillary palp with four palpomeres. Basal palpomere short, the three basal palpomeres with long setae. Apical palpomere cone-shaped with trichoid sensilla, papilliform receptors and slit-like receptors extending to middle area, apically embedded in soft pad.

**Figure 13:**
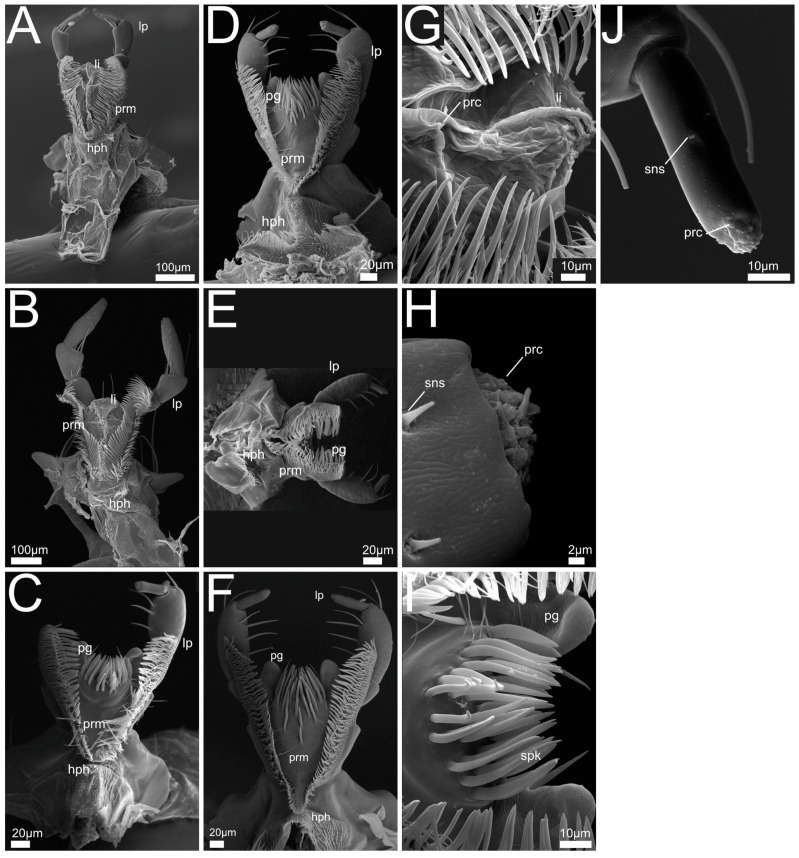
SEM views of Xantholinini, Othiini and Stilicina labium-hypopharynx. Images **A**–**F** dorsal aspect of labium-hypopharynx, **G**–**J** with detailed views of apical palpomeres, ligula and receptors, (**A**) *Gyrohypnus fracticornis*; (**B**) *Othius punctulatus*; (**C**) *Rugilus erichsonii*; (**D**) *R. mixtus*; (**E**) *R. orbiculatus*; (**F**) *R. rufipes*; (**G**) *G. fracticornis*, dorsal aspect anterior prementum and ligula; (**H**) *O. punctulatus*, apical end of labial palp; (**I**) *R. erichsonii*, dorsal aspect of anterior prementum and ligula; (**J**) *R. rufipes*, apical segment of labial palp. Abbreviations: hph: hypopharynx; li: ligula; lp: labial palp; pg: paraglossae; prc: papilliform receptor; prm: prementum; spk: spike-like sensilla; sns: sensilla.

Labium-hypopharynx (Figure 13A,G): Prementum with longitudinal dorsal fold and papilliform receptors along anterior margin. Lateral margin with many mesad- and dorsad-directed spines and hair-like trichomes, converging proximally.

Palps directed distad and with three palpomeres. Second palpomere with distal setae, apical palpomere with papilliform receptors. Ligula of prementum in between antero-lateral lobes as medial unpaired projection equipped with papilliform receptors.

Prementum separated from hypopharynx by transverse suture. Anterior part of hypopharynx with two lateral groups of mesad-directed hair-like trichomes. Posterior part of hypopharynx smooth.

#### 3.1.5. *Othius punctulatus*

Front leg (Figure 5B,H): Claws symmetrical, curving ventro-mediad, with paired long setae extending laterad from empodium. With five tarsomeres, 1–4 with medial tenent setae with spatulate tips, laterally with long unmodified setae, tarsomere 5 elongated and without tenent setae. End of tibia with long spur, and several shorter spurs and comb-like structures directed distad.

Labrum (Figure 7B,H): Free, connected to clypeus by clypeolabral suture. Anterior margin rounded and divided medially with deep cleft, anteriorly with long setae and two rows of distad-oriented shorter setae beginning laterally and converging proximad towards cleft, all setae curving slightly mediad. Anterior epipharynx with medial and lateral paired groups of mesad-directed and anteriorly directed hair-like trichomes, medial area covered with tooth-shaped mechanoreceptors.

Mandible (Figure 9B): Long and conical. Mandibular apex acute and oriented mesad. Incisor area acute, connecting apex with retinaculum. Subapical tooth absent.

Retinaculum developed as two tooth-like projections proximal to incisor area. Lobe-like prostheca developed as a flexible brush with hair-like trichomes, oriented distad. Mola absent.

Maxilla (Figure 11B): Cardo transverse. Stipes subdivided into basi- and mediostipes, the latter forming the base of both lacinia and galea. Lacinia extending to base of galea, fused with mediostipes, apico-mesally differentiated as robust brush with mediad-curving and in the basal region distad-oriented setae. Galea at distal margin of mediostipes, reaching second palpomere, apically differentiated as robust brush with mediad-curving setae. Palpifer slender medially fused with medio- and basistipes. Palpi with four palpomeres, basal palpomere short, setae on basal three palpomeres. Apical palpomere conical with papilliform receptors and apical receptor bundle with campaniform receptors and trichoid sensilla.

Labium-hypopharynx (Figure 13B,H): Prementum with longitudinal dorsal fold and papilliform receptors along anterior margin. Lateral margin with two rows of mesad- and dorsad- (on the outside laterad) directed spines and hair-like trichomes, rows converging proximally.

Palps are directed distad, three palpomeres, second palpomere basally with short setae, medio-apically with longer setae. Apical palpomere with papilliform receptors, and apical receptor bundle with papilliform receptors and trichoid sensilla. Ligula of prementum in between antero-lateral lobes as medial unpaired projection equipped with papilliform receptors. Separation towards hypopharynx by transverse suture. Anterior hypopharynx with lateral tufts of hair-like trichomes, directed mesad. Posterior hypopharynx with posteriorly directed hair-like trichomes, medial area smooth.

#### 3.1.6. *Rugilus* spp.

Front leg (Figure 5C–F,I): Claws symmetrical, curving ventro-mediad, with paired long setae extending laterad from base. Five tarsomeres, 1–4 medially with tenent setae with spatulate tips, laterally with long, unmodified setae. Tarsomere 5 with many, always unmodified setae and elongated. Distal margin of tibia with several small spurs and comb-like structures directed distad.

Labrum (Figure 7C–F,I): Free, connected to clypeus by clypeolabral suture. Anterior margin protruding laterad, medially with emargination and two short setae originating from epipharynx, directed anteriorly (*R. rufipes* Figure 7F) or short spur with long mediad curved setae, directed distad and paired transverse rows of short setae, directed distad and mediad (*R. erichsonii*, Figure 7C, *R. mixtus*, Figure 7D, *R. orbiculatus*, Figure 7E). Anterior epipharynx medially with longitudinal groove, flanked by mesad-directed and anteriorly directed hair-like trichomes.

Mandible (Figure 9C–F): Long and crescent-shaped, mandibular apex acute, oriented mesad. Incisor area straight and connecting apex with retinaculum, subapical tooth absent. Retinaculum developed as three teeth proximal to incisor area. Both prostheca and mola absent.

Maxilla (Figure 11C–F,H–J): Cardo transverse. Stipes subdivided into basi-and mediostipes, the latter forming the base for galea and lacinia. Galea at apex of mediostipes. Galea extending up to second palpomere of maxillary palp. Galea apically differentiated as robust brush with slightly mediad-curving setae. Lacinia apico-mesally differentiated as a robust brush with mediad-curving setae, reaching galea. Palpifer slender, medially fused with basistipes, only distal margin reaching mediostipes. Palp with four palpomeres, basal palpomere short, third palpomere slightly bulbous, apical palpomere small and conical. Second and third palpomeres with setae, apical palpomere with papilliform and slit-like receptors, stretching from proximal margin of apical palpomere to medial region (no such receptors visible in *R. orbiculatus*, Figure 11E,I, possibly because of dirt or smaller size).

Labium-hypopharynx (Figure 13C–F): Lateral margin of prementum with many mesad- and dorsad-directed spines and hair-like trichomes, converging proximally. Palps directed distad, three palpomeres. Second palpomere bulbous, medial with long mediad-directed setae, apical palpomere slender, parallel-sided and small, apically with campaniform receptors and soft pad (Figure 13D). Prementum with antero-lateral lobes and additional medial paired lobes (probably paraglossae) directed distad, lateral of a tuft of distad directed spike-like sensilla (Figure 13I) (probably fused glossae).

Separation towards hypopharynx by transverse row of trichomes and spines and a transverse suture. Anterior hypopharynx with anteriorly directed hair-like trichomes. Hypopharynx proximally delimited by transverse row of anteriorly directed hair-like trichomes (*R. mixtus*, Figure 13D).

#### 3.1.7. Mandible Shapes

**Figure 14:**
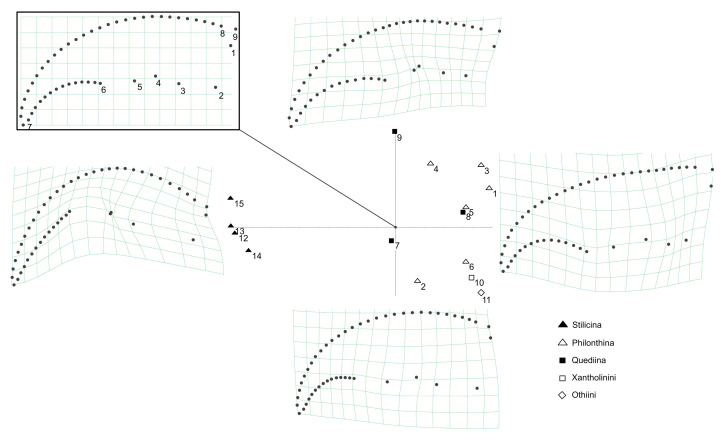
Principal component analysis of nine landmarks and 45 semilandmarks for 15 species. Species sorted by (sub-)tribe (see legend). PC1 along the *x*-axis, PC2 along the *y*-axis. For species numbers, see Table 1. Warped mandibles are depicted at the end of each PC, reference mandible (numbers correspond to landmarks in Figure 1) depicted at top left corner.

The relative warp analysis of the mandible shapes revealed two notable principal components referred to as PC1 and PC2 that cumulatively explained 83.1% of the total variance. PC1 (explaining 68.5% of total variance) is connected with a slight decrease in curvature of the mandible so that it becomes straighter and more rectangular at the base. Towards its negative direction, PC1 is associated with a strong bend of the apex and incisor area in relation to the base of the mandible, and a decrease in the width of the mandible, causing the mandible to become more sickle-shaped compared with the reference mandible (Figure 14). Towards the positive direction, PC2 (explaining 14.6% of total variance) is associated with a lengthening of the incisor area and a narrowing of the mandible, causing it to become more falciform than the reference mandible. Towards the negative direction of PC2, the incisor shortens and the mandibular base widens, causing the mandible to become more robust (Figure 14). Species of the genus *Rugilus* (subtribe Stilicina) form a cluster at the left end of the PC1 axis, meaning that their mandibles are sickle-shaped and narrow. With respect to the PC2 axis, the Stilicina cover only about a third of its total variation while being located in the intermediate range, meaning that the relative length of the incisor area compared with the base of the mandible is intermediate. The investigated species of the tribes Staphylinini (Philonthina, Quediina), Xantholinini, and Othiini accumulate along the right-side end of PC1, being indicative of mandibles with an overall wider shape in comparison to the sickle–shaped mandibles at the negative end of PC1. These clades cover the entire range of PC2. Both considered species of the tribes Xantholinini and Othiini (No. 10 and 11 in Figure 14) are found in the bottom right area, i.e., their mandibles are wide and robust with a relatively short incisor. The species *Philonthus rufipes, P. marginatus*, and *Quedius cinctus* (No. 3, 4 and 9 in Figure 14) are found at the positive end of PC2, i.e., their mandibles have a long incisor and are falciform (especially *Q. cinctus* and *P. marginatus*). Pairwise Mahalanobis distances between (sub-)tribes with resulting *p* values of the CVA calculated to quantify the separation between the (sub-)tribes are shown in Table 2.

#### 3.1.8. Group Predictions Using Morphological Ratios and Landmark Measurements

In the stepwise Discriminant Function Analysis (DA) performed to detect any variables discriminating between the different tribes, only the variables of head length/pronotum length (*p* < 0.001) and geometric morphometric PC1 significantly (*p* < 0.001) contributed to group separation (for values of original data see Appendix A Table A1).

**Figure 15:**
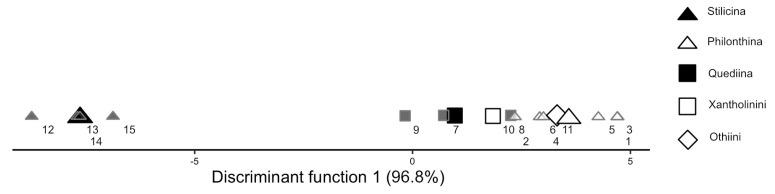
Plot of linear discriminant analytical separation of the tribes. Small transparent shapes represent individual species, large dark shapes represent group centroids. Only one species was analysed for Othiini and Xantholinini. The data (Appendix A, Table A1) were log10-transformed prior to the analysis. Only the first discriminant function, separating Stilicina from the Quediina and Philonthina, is plotted. It shows the following standardized canonical discriminant coefficients: head length/pronotum length: −0.60; PC1: 0.76.

Only the first discriminant function significantly contributed to the separation of the tribes, separating Stilicina from the Quediina plus Philonthina and explaining 96.8% (Wilks’ lambda = 0.043; eigenvalue = 33.36; canonic correlation: 0.99) of total variation (Figure 15).

### 3.2. Predatory Behaviour

The predatory behaviour of the beetles under study was previously briefly described [47] and is addressed in greater detail in the following. Table 3 summarizes in which species the respective behaviours were observed.

#### 3.2.1. Prey Detection

**Figure 16:**
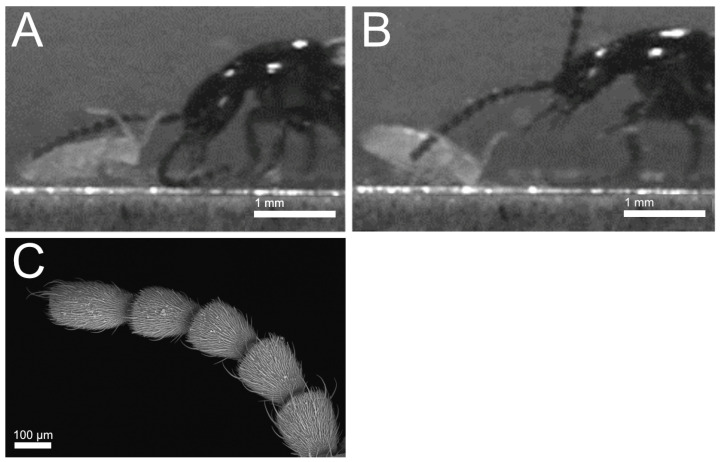
*Philonthus varians*. Subliminal touching of a *Heteromurus nitidus* springtail by thin setae on the antennae of the beetle, but without triggering the springtail’s escape response (lateral view, **A**,**B**). SEM view of antenna (**C**).

In all the beetle species examined, representatives were observed touching the highly sensitive springtails, subliminally to their flight response, with their antennae (Figure 16). The beetles appear to have very thin and soft sensory setae emerging from each segment of their antennae (Figure 16C). They use these bristles to sense mechanically the position of the springtails without alarming them.

#### 3.2.2. Prey Seizure

Four different prey-capture techniques were detected in the investigated species.

**Figure 17:**
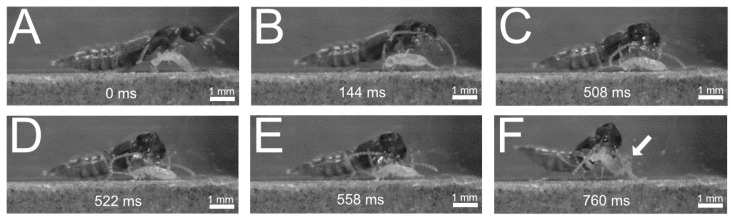
*Rugilus rufipes*. Direct seizure with the mandibles (lateral aspect). Time course of depicted sequence is provided in milliseconds elapsed from the start (= image **A**). Images **B**–**F** depicting the process of prey capture. For further description, see text. Arrow in **F** points to front leg. Source: [47].

(1) Direct seizure with the mandibles (Figure 17)

During the slow approach towards the prey, the mandibles are opened without being detected by the prey. The actual attack follows at high speed, whereby the beetle hurls its body forward and rapidly closes its mandibles to fix the prey (Figure 17D,E). While pushing forward, the beetles usually move their antennae backwards (Figure 17E). Finally, the prey is grasped by the mandibles and the front leg pair can also be used to position the food optimally (arrow in Figure 17F).

**Figure 18:**
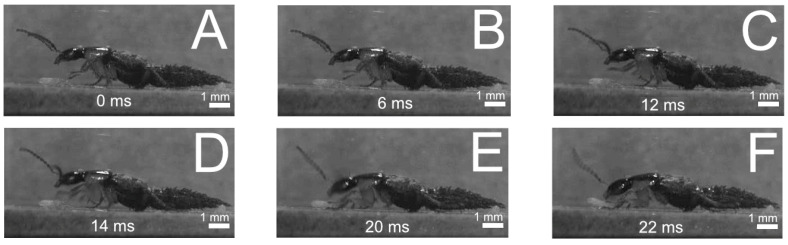
*Philonthus marginatus*. Predatory strike with the front legs (lateral aspect). Time course of depicted sequence is provided in milliseconds elapsed from the start (= image **A**). Images **B**–**F** depicting the process of prey capture. For further description, see text. Source: [47].

**Figure 19:**
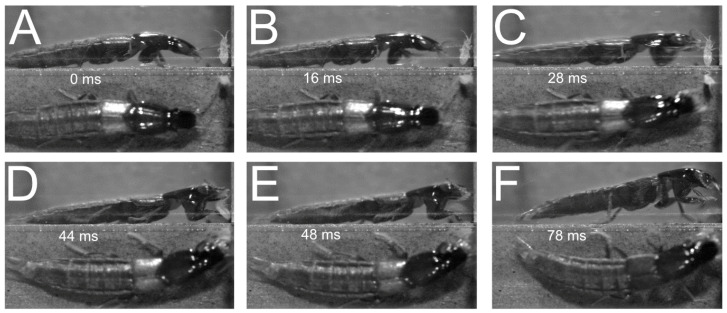
*Quedius curtipennis*. Overwhelming of a *Heteromurus nitidus* springtail by using the front pair of legs in the initial attack. In the last image of the sequence, the initiation of caging behaviour is visible after the springtail escaped the initial attack (upper parts: side view; lower parts: top view). Time course of depicted sequence is provided in milliseconds elapsed from the start (= image **A**). Images **B**–**F** depicting the process of prey capture. For further description, see text.

(2) Predatory strike with the front legs (Figure 18 and Figure 19)

The behaviour conducted with the front legs was described for *Philonthus marginatus* by [18]. The front legs are regularly held in an alert position (Figure 18A). Once the prey approaches the beetle, the beetle moves its front legs above the prey (Figure 18A–C) and strikes them down rapidly onto the prey (Figure 18D,E) followed by the final seizure and feeding via the mouthparts (Figure 18F).

A similar behaviour was observed in *Philonthus varians* and *Quedius curtipennis* (Figure 19) when hunting springtails. Similar to *P. marginatus*, *Q. curtipennis* beetles were observed to keep their front pair of legs in an alert position off the ground (Figure 19B), but only when prey was close. When it rapidly moved its front legs onto their prey, the beetle rushed forwards with its whole body onto the prey in order to grab it immediately with its mandibles, the front legs reaching the prey simultaneously (Figure 19A–C).

**Figure 20:**
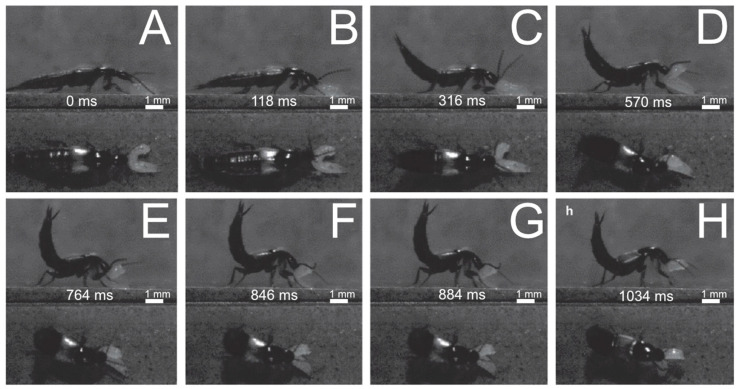
*Philonthus varians*. Pulling, i.e., direct seizure with the mandibles followed by lifting and dragging the prey backwards (lateral and dorsal aspects). Time course of depicted sequence is provided in milliseconds elapsed from the start (= image **A**). Images **B**–**H** depicting the process of prey capture. For further description, see text. Source: [47].

(3) Pulling backwards (Figure 20)

Direct seizure with the mandibles is followed by the lifting and dragging of the prey backwards. Once the beetle grasps the prey with its mandibles (Figure 20B), it starts pulling the prey backwards (Figure 20C). During this process, the antennae might be moved backwards and the highly movable abdomen upwards (Figure 20C). The beetle pushes the front part of its body upwards, with the prey continuously being grasped by its mandibles (Figure 20D). In continuation of the pulling movement, the beetle walks backwards, still holding the prey in its mandibles (Figure 20D–G). The antennae are kept retracted during this phase. The prey is finally held up off the ground (Figure 20G,H) and then seized with the mandibles and the other mouthparts.

**Figure 21:**
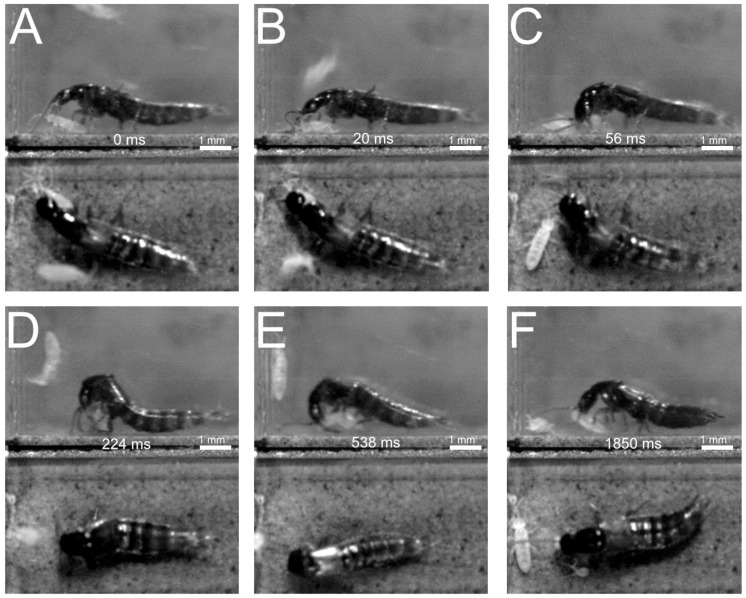
*Bisnius sordidus*. Formation of a catching basket, i.e., shoving the prey (with the head, the mandibles and/or the front legs) beneath the pronotum and between the inner sides of the front legs to form a cage-like structure that encloses the prey (lateral and dorsal aspects). Time course of depicted sequence is provided in milliseconds elapsed from the start (= image **A**). Images **B**–**F** depicting the process of prey capture. For further description see text. Source: [47].

(4) Formation of a catching basket (Figure 21)

Here, the prey is manoeuvred (by the head, the mandibles, and/or the front legs) beneath the thorax, with the inner sides of the front legs that together form a cage-like structure enclosing the prey (Figure 21). The beetle approaches the prey, shifting its body cautiously above the prey (Figure 21A,B), bending its head down and manoeuvring the prey beneath its thorax, resulting in the springtail finally being enclosed by the legs and the body of the beetle (Figure 21C). Some beetles that use this same hunting technique were observed to perform first a grip either with the mandibles or, in *Philonthus marginatus*, with their front legs, in order to manoeuvre the prey beneath their body [18]. All pairs of legs are finally involved in enclosing and fixing the prey, while the beetle continues to bite the springtail with its mandibles (Figure 21D,E). In some cases, the beetles fell on their side or back during this process, with the prey enclosed by all pairs of legs. At some point, the beetle stops enclosing the prey with all pairs of legs and returns to its initial position, still seizing the prey with its mouthparts and readjusting its position with the front pair of legs (Figure 21F).

#### 3.2.3. Positioning with Front Legs While Feeding

In representatives of both subfamilies, the front pair of legs was often observed to position the prey in the feeding procedure that followed a successful prey-capture event (cf. Figure 17F, Figure 20E and Figure 21F; Table 3). While doing so, the front legs moved constantly in asynchrony with each other, similar to walking movements or to the use of the front legs during the cleaning of the head and the antennae. The movement itself was conducted from a dorso-lateral position beside the head downwards, reaching to medial regions beneath the head and the thorax, as if the beetle was beating a drum. While the beetle was feeding, the prey was killed probably by the physical damage that also resulted from this process. If the prey was strongly moving, it was operated upon with the front legs as described above and crushed by the mandibles until the movement stopped.

#### 3.2.4. Typical Sequences of the Observed Prey-Capture Patterns

**Figure 22:**
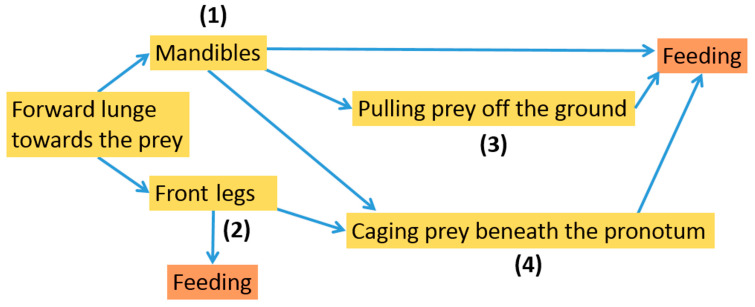
Flow diagram illustrating the inferred pathway of observed prey-capture patterns with their possible combinations and sequences. Source: [47].

The behaviours described so far were found to be combined in various ways (Figure 22). The prey was attacked directly after the beetles had lunged forward to the prey, by either (1) the mandibles or (2) the front pair of legs. Both cases led directly to feeding. Alternatively, an attack with the mandibles led to (3) the pulling of the prey off the ground (often by walking backwards) before feeding. After the attack was performed with either the mandibles or with the front pair of legs, (4) the prey was caged underneath the thorax followed by being eaten (Figure 22). Beetles were also observed that used their entire head capsule for shoving the prey underneath their body, instead of first attacking it with their mandibles or front legs.

#### 3.2.5. Occurrence of the Behaviours Observed in the Examined Species

Table 3 lists those species that were observed carrying out the specific types of behaviour.

Attacking the prey with the mandibles, dragging the prey, positioning the prey with the front legs, mechanical touching of the prey with the antennae, and caging it with the legs are behaviours that were observed within every subfamily of the sample. The caging behaviour was observed in all representatives of each tribe and subtribe present, except for the tribe Othiini with its only representative *Othius punctulatus* in this study (Table 3). The attack being initiated by the front legs was observed not only in *Philonthus marginatus* [18], but also in *P. varians* and *Quedius curtipennis* (Table 3).

#### 3.2.6. Relationships between Morphological and Behavioural Traits

**Figure 23:**
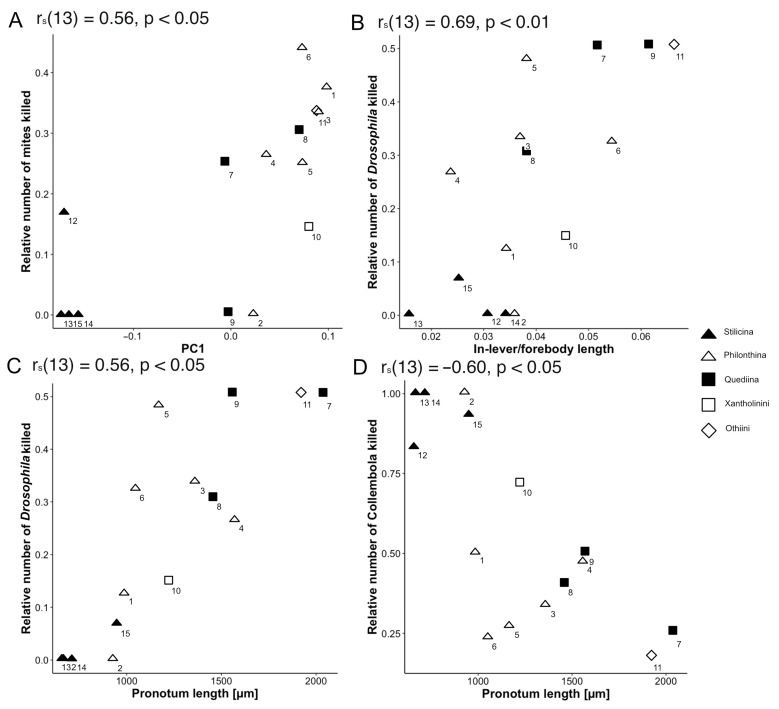
Plots of untransformed original data with Spearman correlations (r_s_) for PIC-transformed variables. Relationships between (**A**): PC1 and the relative number of mites killed, (**B**): In-lever/forebody length and the relative number of *Drosophila* killed, (**C**): pronotum length (µm) and the relative number of *Drosophila* killed, (**D**): pronotum length (µm) and the relative number of Collembola killed. Numbers correspond to species abbreviations in Table 1.

For PIC-transformed data, significant positive Spearman correlations (Table 4) were found for several body-length-related measurements and the relative number of *Drosophila* killed, e.g., pronotum length (r_s_(13) = 0.56, *p* < 0.05) (Figure 23C) and in-lever/forebody length (r_s_(13) = 0.69, *p* < 0.05) (Figure 23B, for untransformed data, see Appendix A, Table A1, including predatory performance towards the three prey types). An additional positive correlation was found between PC1 of the geometric morphometric analysis of the mandible shape and the number of mites killed (r_s_(13) = 0.56, *p* < 0.05) (Figure 23A). A negative significant correlation was found only for pronotum length and the relative number of collembolans killed (r_s_(13) = −0.60, *p* < 0.05) (Figure 23D).

## 4. Discussion

Because of their tremendous ecomorphological diversity and worldwide distribution, rove beetles have become an increasingly investigated group of insects in ecology and evolution (e.g., [3,5,24]). In contrast to phytophagous insects, rove beetles have mainly diversified through their various ways of living in the litter layer. In the current contribution, we focus on some representatives of the subfamilies Paederinae and Staphylininae, which comprise modern, mostly predatory, life forms [4,5,48]. To improve our understanding of patterns and trends in the evolution of the (functional) head morphology of modern Staphylinidae, we studied their mouthpart morphology in concert with the predaceous feeding behaviour in beetles of selected species and interpret the ways in which the mandibles, in particular, are linked to prey-capture performance. We followed a three-step approach: (1) a comprehensive SEM analysis of the mouthparts (integrating geometric morphometric analyses of the mandible shapes) and the front legs, (2) highspeed videography of prey-capture behaviour, and (3) phylogenetically independent statistical analyses to reveal any potential links between morphology and prey-capture performance. Our study involved an exploratory approach, i.e., our taxon sampling did not follow a systematic approach (e.g., covering major clades within the “staphylinine group”, sensu [49]) but depends on the species that we collected within a certain area, habitat, and time frame connected to our study site. We found interesting insights into the relationships among morphology, behaviour, and prey-capture performance that make further exploration of this topic in staphylinids a promising area of research.

Most representatives that we studied belong to *Philonthus* and *Quedius* (both Staphylininae: Staphylinini), which we compared with beetles of the genus *Rugilus* (Paederinae: Paederini). Adults and larvae of the investigated clades comprise modern predators, many have evolved complex mouthpart modifications that are correlated with their highly specialized feeding and preoral digestion (e.g., [5,50]). The results of our study confirmed previous findings [18] suggesting that, in addition to the mouthparts of the beetles, their front legs are involved at least in the positioning of their prey during feeding. Therefore, we also investigated the foretarsi in more detail.

### 4.1. Morphology

Front legs: With the exception of *Bisnius sordidus* (Philonthina) and *Gyrohypnus fracticornis* (Xantholinini), we found especially widened foretarsomeres equipped with tenent setae having obviously increased adhesive properties in most representatives of the genera *Philonthus, Quedius, Othius,* and *Rugilus*. This was found for both, male and female specimens. Our observations of predatory behaviour, with two *Philonthus* and one *Quedius* species employing their front legs for prey seizure, suggest that such specialization might be connected to predation rather than to other biological contexts such as mating. A potential scenario might be that the front legs were ancestrally involved in the positioning and manipulation of the prey during the feeding process and have later become increasingly involved in the prey-capture process, as previously observed in *Philonthus marginatus* [18].

Labrum-Epipharynx: Generally, in all the examined species, the anterior margin of the labrum is equipped with long anteriorly directed setae that probably play a role in mechanically sensing the seized prey; this is consistent with descriptions of staphylinid labra in previous studies [51]. Whereas, in the investigated Staphylinini, the surface of the epipharynx is densely covered by mediad- and forward-directed hair-like trichomes (probably absorbing digested fluid from preoral digestion and/or preventing solid material from passing into the pharynx [8]); members of both the Xantholinini and Othiini show a conspicuous field of specialized mechanosensilla hinting at a special mode of feeding (and potentially food items) in these beetles. In *Rugilus* (Paederinae), the epipharynx is characterized by a prominent medial groove bordered by fringes of antero-mediad-directed hair-like trichomes. The groove might help the beetle to efficiently imbibe and channel the preorally digested fluid (including haemolymph) from the exterior towards the pharynx. Alternatively, it may reflect different food choices that require less filtering than the food consumed by Staphylinini or a higher tolerance for solid material.

Mandible: In contrast to the ground-plan of microphagous mouthparts in staphylinoid adults [26], the mandibles of all the beetles under study lack a basal mola. This is in accordance with their predatory lifestyle in combination with preoral digestion that does not require any crushing or grinding of fine particulate material. This reduction was also described for other predatory staphylinids [51]. A brush-like prostheca can be found in almost all the examined clades. Since this feature belongs to the microphagous groundplan in Staphylinoidea [26], its presence suggests that these beetles have a mixed-feeding strategy (including food material additional to obligate predation on living animals), although this structure might alternatively have experienced a change in function and is now used for more effective gathering and concentrating of the pre-orally digested fluid originating from the prey. In *Rugilus* spp., the prostheca is absent, a characteristic that distinguishes these beetles as more specialized predators. Other specialized staphylinid predators such as *Stenus* spp. also lack a mandibular prostheca [10]. Another feature present in all the investigated species are the retinacula, i.e., the mesally directed teeth of the middle mandibular region not integrated into the incisor area [26,52]. Depending on the species, such teeth occur in numbers between one and three. They are not constituents of the microphagous groundplan in Staphylinoidea [26] and are especially pronounced in *Rugilus*, both of which findings are indicative of their potential function in supporting prey seizure. In *Gyrohypnus fracticornis*, we found an additional subapical tooth that lies dorsal to the incisor area and is not present in the other investigated species, the function of this tooth is presently unclear. The pronounced predatory specialization of the *Rugilus* mandibles is further supported by our geometric morphometric shape and discriminant function analyses. Here, *Rugilus* spp. differ from the other clades in their more falcate appearance and the more strongly tapered incisor that might facilitate the piercing of their prey. Moreover, in our geometric morphometric shape analysis, Philonthina and Quediina show some variation along the relative warp axis 2, which distinguishes between the more robust and broad mandibles and the more delicate and falciform mandibles. More robust and forceful mandibles as indicated by higher PC1 scores and higher relative in-lever lengths in our (geometric) morphometric analyses might be considered adaptations towards more robust and mechanically resistant prey, as indicated by the positive correlation between this mandible shape and prey-capture success towards hard-shelled mites and relatively robust *Drosophila* maggots (cf. Figure 23A,B).

Maxilla: The maxillae of all the investigated species retain a rather conservative structure. This largely corresponds with the microphagous groundplan features in Staphylinoidea [26] and the maxillae described for related staphylinids [53], i.e., both galea and lacinia bearing mediad-directed brush-like structures that are well-suited to sweep in all kinds of food material and to keep the prey in place during mandibular kneading and preoral digestion.

Labium-hypopharynx: Similar to the maxillae, the labium-hypopharynx represent the overall ancestral ground-plan features in Staphylinoidea [26], featuring a medial longitudinal groove (“bristle-trough”) that is bordered by hairs or spines. This formation is generally suited to concentrate the food stream in the midline and to channel it towards the mouth opening. In the investigated members of the Staphylininae, the medial part of the ligula forms a lobe-like projection covered by papilliform sensilla. In the paederine *Rugilus* spp., the anterior prementum shows a more complex organization. Here a prominent tuft of anteriorly directed spike-like sensilla (probably the glossae) that might play a special role in prey-detection and manipulation lies between the medial paired lobe-like projections, that probably represent the paraglossae.

### 4.2. Predatory Behaviour and Performance

In insects, predation consists of four successive phases [22]: (1) prey search, (2) prey recognition, (3) final attack, and (4) seizure while eating the prey. The sensory organs, frontal body parts and mouthparts are adapted to the efficient grasping of the prey in at least one of these phases [22]. A functional understanding of the morphology of the parts involved in the foraging process requires behavioural observations. After active or passive (sit-and-wait) search and detection, proper recognition of the prey is essential. In this phase, visual, chemical or mechanical clues help the predator to recognize the prey and estimate its exact location [22]. While approaching and recognizing the prey should be subliminal as far as the prey is concerned, the actual attack might occur at maximum speed and acceleration [22]. Various means can prevent the prey from fleeing and involve principles such as cutting off the escape route of the prey, being faster than the prey, or the performance of unexpected thrusts [22]. Cutting off the escape route, for example, is a strategy used by the carabid beetle *Loricera pilicornis* Fabricius, which hunts springtails by enclosing them underneath its two antennae and by forming a cage around the springtails with its long and stable bristles [54]. A good example for an approach involving excess speed is shown by *Stenus* beetles, which are capable of extending their protrusible labium and catching springtails within 3–5 milliseconds [11,24], which is much faster than the 20–50 milliseconds needed by a springtail to jump off the ground [55,56]. For the seizing action, mouthparts with sharp mandibles are used for biting and chewing, whereas brush-like or spiny maxillae keep the prey in position. Some beetles within the Pselaphinae have been described to feed while holding their prey in place with the additional help of their front legs [5,57,58]. This has also been observed within the Staphylininae [19,20]. Other staphylinid beetles, e.g., several pselaphines [57,58], and the Staphylininae *Philonthus marginatus* [18] and *Nordus fungicola* [59], use their front legs for a predatorial strike. Preoral digestion (often in connection with “rotary mill” behaviour [6]) is also a common phenomenon among Staphylinidae, which add enzymes to their chewed prey and pump the resulting digested liquid into their intestinal tract [5].

Although beetles of the two subfamilies, Staphylininae and Paederinae, have been well-studied at the morphological level, little is known about their actual predatory behaviour. However, behavioural observations to correlate the studied morphological properties with the actions performed by the examined structures are mandatory if solid conclusions concerning their function are to be gained. We therefore designed an observational behavioural study to investigate the prey capture of representatives of these subfamilies.

### 4.3. First-Step Behaviours and Adjustment Strategies

According to the categorization of [22], palpation of the prey with the antennae can be considered a behaviour that serves the location and recognition of prey prior to an attack. Mechanical touching of the prey with the purpose of the spatial recognition of its exact position and size needs to be undertaken without detection by the prey. In terms of prey seizure, four different behaviours can be distinguished that involve not only the mandibles, but also (in some species) the front legs. These behaviours can be subclustered into two categories: (1) first-step attacks (performed prior to any other behaviour), (2) and adjustment strategies. First-step attacks involve the direct attack of the prey and lead to its direct fixation. They are performed quickly, are unnoticed by the prey and are directly followed by feeding. The adjustment strategies involve the positioning of the prey with the front pair of limbs, behaviour that has been found in the beetles of all the investigated species and that might be considered a pre-stage to the evolution of true predatory legs such as those present in *Philonthus marginatus* [18]. Since the prey is alive during feeding and seems to be killed mainly by physical damage, the beetles need to avoid the prey escaping during the early stage of feeding and to keep the prey in a suitable position during the remaining feeding process. Most of the pselaphine beetles investigated by [58] lifted the front part of their body after the strike and manipulated “the prey with their front legs (tibiae and tarsi), while the middle and hind legs ensured a firm stance”. This behaviour resembles the prey positioning observed in *Stenus* beetles [7] (Figure 3) and is also performed by the Staphylininae and Paederinae beetles that we observed, but with the difference of a much steeper angle of the body axis towards the ground compared with Pselaphinae because of the latters’ more compact and therefore less flexible morphology. Dragging the prey and caging it beneath the thorax between all pairs of legs (see also [18], Figure 2) enable the beetle to adjust its prey-handling after a first-step attack. The two events are often initiated in special situations occurring in the context of overwhelming prey that shows special flight or defence mechanisms. Dragging of the prey has most frequently been observed towards soft *Drosophila* larvae, a prey that resembles worms and worm-like animals living in the substrate. In such prey types, the predator is confronted with the prey being strongly attached to the substrate and strongly moving when it becomes detached. Hence, detaching the prey from the substrate by lifting it upwards, while walking backwards, increases the capture success attained by the predator. The retraction of the antennae during dragging prevents their possible damage by any strong defence movements of the prey when it is detached from the substrate. While dragging, the beetles often lift their highly movable abdomen, which might help them to counterbalance the drastic movements of their prey.

The capture of fast-fleeing prey such as springtails, or in other habitats, flying insects is completely different from the situation described above. This type of prey specializes in fleeing as quickly as possible and as soon as a potential threat is detected. Here, predators can increase their success in the hunt either by fixing their prey upon contact, immediately before its flight response is fully performed, or by cutting off its escape route. The observed behaviour of manoeuvring the prey beneath the body of the beetle and enclosing it between all pairs of legs after a first-step attack is certainly one strategy for cutting off an escape route of the prey and thereby for increasing the success of the beetle during their hunt for fast-fleeing prey such as springtails. This behaviour was observed in the investigated representatives of both Paederinae and Staphylininae suggesting its early evolution in the stem group of both these clades. In the distantly related subfamily Pselaphinae, similar behaviour was previously observed for the handling of springtails after a predatory strike; they were described as “holding the prey sandwiched between tibiae and femorae” [58]. This resembles the behaviour described in our investigation except that, in the observed Staphylininae and Paederinae, the hind pair of legs was also used to enclose the prey. This sometimes led to the beetle falling over and lying on its back, with its prey enclosed between all pairs of legs.

The lifting and dragging of the prey and its enclosure under the body of the beetle by all pairs of legs was observed to be modifiable and combinable in various ways. These findings lead to the conclusion of situational adaptability to the respective prey type and their response. They also indicate the important role of mechanical stimuli in the recognition of the prey type and its escape behaviours. Questions now arise as to which prey parameters induce the initiation of which behaviours and whether the beetles’ behaviours can be adapted by learning and/or an inherent program reacting to fine stimuli during situational changes. The behavioural adaptions towards the various escape strategies found in Staphylininae and Paederinae (and Pselaphinae [58]) make them comparable with the multifunctional tools evolved by generalist predators in an environment rich in diversity with regard to their small prey arthropods and their various defence mechanisms.

In conclusion, the variable combination of behaviours during a prey-capture event (cf. Figure 22) suggests some behavioural flexibility and adjustability with respect to the specific situation. For example, if the gripping performed in behaviour (1) or (2) does not lead to the proper fixation of the prey, behaviour (4) can be employed.

### 4.4. Potential Specializations towards Certain Prey Types

All observed beetles in this study were capable of touching springtails without triggering their flight response. This indicates special predatory potential towards sensitive and fast fleeing prey such as highly elusive springtails. Such special prey detection capabilities were previously described in adult beetles of the subfamily Pselaphinae [58] and larvae of the tachyporine *Sepedophilus testaceus* [60]. Subliminal mechanical sensing is usually possible with the observed delicate sensory setae emerging from antennal segments or other body parts.

The investigated *Rugilus* beetles seem to be specialized towards elusive prey such as springtails. One indication for this specialization is the specificity of their hunting technique. Their rapid forward attack movements, which surprise their prey, are characteristic for beetle predators that prey on springtails (e.g., [61]). In the observed *Rugilus* species, the final strike occurs within the range of the flight response of springtails, which take 10–50 ms to jump off the ground after receiving alerting signals [55,56]. Therefore, the rapid attack technique and high hunting success towards elusive springtails, as observed in *Rugilus*, suggest that these beetles have adapted evolutionarily towards springtails as preferred prey. This view is further supported by the morphological structures of the mandibles with their long and slender falcate mandibles and their especially low in-lever values (cf. Appendix A, Table A1) enabling them to generate high velocity outputs in the trade-off between force and velocity. Moreover, the characteristically thin *Rugilus* neck might be advantageous in the precise adjustment of the head towards the prey before the final strike.

An especially high preference for springtails was also found in both of the observed *Gyrohypnus* individuals (cf. Appendix A, Table A1). Whereas no special hunting method was visible at first sight, the beetles mainly performed a mandible grip from above (in a relatively slow manner), pressing the springtail onto the substrate while directly gripping it with their short and strong mandibles. This might be indicative of their specialization for this kind of prey. The elongated head, which might function as a dorsal barrier for the springtail, further supports this idea. However, the strong and robust mandibles with their additional subapical tooth are probably especially useful against hard-shelled prey. Indeed, one of the two specimens were observed to crack the hard shell of an oribatid mite. *Gyrohypnus* beetles are sometimes described to cut the escape route of isopods by lying curled around them on their sides before attacking them [62]. Isopods are mostly hard-shelled and, hence, the robust *Gyrohypnus* mandibles might perform well when dealing with this kind of prey.

The *Othius punctulatus* beetles showed the smallest proportion of springtails killed amongst their total prey (cf. Appendix A, Table A1). However, because of the small sample size, no reliable assumptions can be made with respect to their prey specializations. The possible absence of a caging behaviour, which seems to be adaptive towards fast fleeing prey (see below), suggests that these beetles are not specialized for attacking fast fleeing prey. The representatives of this species showed especially strong adhesive power with regard to their tarsal setae when being transferred between the experimental compartments. This might be beneficial in dragging the prey off the substrate. The mandibles of these beetles are short and appear to be strong; they can therefore be used to crack hard-shelled prey such as the presented *Archegozoetes longisetosus* mites.

*Philonthus marginatus* beetles show indications for preferentially hunting springtails; the various morphological and behavioural specializations that they exhibit help them to efficiently capture springtails [18]. Similar to the *Rugilus* beetles, the *P. marginatus* specimens are highly capable and motivated when hunting springtails. However, contrary to *Rugilus*, they successfully and regularly hunt other types of prey. This leads to the conclusion that *P. marginatus* represents a generalist species with only a tendency towards specialization towards elusive prey such as springtails.

No sufficient indications concerning specializations towards a certain type of prey were registered for the other species in our study.

### 4.5. Raptorial Legs in Staphylinid Beetles

Predatory front legs have evolved independently in various clades of insects. These legs resemble each other as a consequence of evolutionary adaptations towards similar optimized functional demands [22]. One of the most prominent examples is the order Mantodea, although raptorial legs have evolved in various heteropterans. They can also be found in Neuroptera within the family Mantispidae and in Mecoptera within the family Bittacidae [22]. Even in Diptera, some species are described to possess raptorial legs [22]. In Coleoptera, to our knowledge, raptorial legs were only described in Staphylinidae, i.e., within Pselaphinae, namely *Tyrus mucronatus, Cedius spinosus*, and *Tmesiphorus costalis* [57,58], and also in *Nordus fungicola* [59] and *Philonthus marginatus* [18], the last two mentioned belonging to the subfamily Staphylininae and the tribe Staphylinini propria as well as in Gyrinidae [63].

Among the observed Staphylinini in the present study, the beetles of three species, *Philonthus marginatus* (as previously described by [18]), *P. varians* (both Staphylinina propria: Philonthina), and *Quedius* cf. *curtipennis* (Quediina), use their front limbs first when attacking prey. *P. marginatus* and *P. varians* are closely related, and so the same route in the evolution of the raptorial use of their front legs is likely. However, *Q.* cf. *curtipennis* is more distantly related to both of these species [37]. In the other studied species of both subtribes, none of the observed beetles employed their front legs for attacking the prey, leading to the assumption of parallel selection of the predatory use of the front legs of *P. marginatus* and *Q.* cf. *curtipennis*. The observed differences in the predatorial striking technique between *Philonthus* and *Quedius* suggest that the beetles in *P. marginatus* and *P. varians* individuals strike with their front leg/s first and grip their prey (if caught) with their mandibles only later, whereas *Q.* cf. *curtipennis* individuals reach out to their prey with the whole front part of their body, moving towards the prey with their mandibles and with the extended tarsi of both front legs being simultaneously held out on both sides of the head. Further comparative research (including the morphological traits involved) is needed to draw final conclusions from these interpretations. Similar observations will probably be made for other species of *Philonthus* and *Quedius* (and Staphylininae), if a broader taxon sample is examined. One feature that all the observed beetles had in common was their handling of the prey with both the front legs while they ate it. The movement of the front limbs during this behaviour resembles the movement of the hunting strike of *P. marginatus* and *Q.* cf. *curtipennis* beetles with regard to the described ‘drum beating movement’ of the front legs. One of the main triggers that evokes this behaviour seems to be a nearby food source. Other behaviours shared by the beetles with raptorial legs in our represented groups of beetles are the lifting/dragging and caging behaviours suggesting that they were previously part of the behavioural repertoires and strategies of their common ancestors. One character of the dragging behaviour is the lifting of the thorax and head of the beetle leading to a high-backed position resembling the resting posture of *P. marginatus* and *Q. curtipennis*. Thus, the lifting and dragging behaviour can be viewed as potential pre-stages that promoted body postures connected to hunting with raptorial legs (cf. [18], Figure 1a).

### 4.6. Relationship between Predatory Performance and Morphology

We used non-parametric correlation analyses of the phylogenetic independent contrasts of the means of each morphometric and predatory performance variable (cf. Appendix A, Table A1) to reveal any connections between these two aspects. Our analyses revealed that larger species perform more successfully towards *Drosophila* maggots, but less successfully towards springtails. In the context of prey capture, body size can be considered a proxy for the overall physical strength and persistence of a beetle, both of which are obviously required for the beetle to overwhelm a violently wriggling drosophilid larva. The significance of force (rather than velocity) for subduing this prey type is further supported by the positive influence of the relative mandibular in-lever length on predatory success on *Drosophila* maggots.

On the other hand, the hunting of rapidly moving prey such as springtails, which are capable of rapid and unexpected escape responses, requires considerable physiological and locomotory agility that is obviously better achieved in the smaller species among the investigated species. Indeed, in his comparative analyses of prey-capture success in staphylinid *Stenus* beetles [64] found that the highly agile bare-ground dwellers among the species under study performed much better in grasping large elusive springtails with the mandibles than the less agile (more clumsy) representatives that forage in plant debris and/or vegetation.

Another correlation involves the mandible shape as represented by the first relative warp (cf. Figure 14) in our geometric landmark analysis. We found that more robust mandibles perform better in successfully overcoming hard-shelled oribatid mites. The special features of the mandibles that are used for breaking the shells of these mites remain unclear but the protruding blunt area of the retinaculum between the landmarks 5 and 6 (e.g., Figure 9A,B) might function as a crushing shear for this type of prey.

## 5. Conclusions

The form-function-performance paradigm (e.g., [65]) in ecomorphology proved to be successful in demonstrating the way that ecologically relevant performance patterns, such as feeding or running, interact with the underlying morphology of the involved body structures, such as head morphology or leg structures [66]. Although the study of ecomorphology seeks to elucidate the overall functional and biomechanical connections between morphology and performance (keyword: ecomechanics), it should also explain the ecomorphological disparity of a clade based on mechanistic explanations with regard to morphological structures that form direct interfaces to the environment.

The present contribution presents a promising approach within the study of ecomorphology by comparing the morphology of mouthparts and front legs of selected species (from two related rove beetle subfamilies) and setting this aspect in relation to predatory behaviour and predatory performance towards three types of prey. Although our study follows an exploratory approach that examined species collected within a limited study area and within a limited time frame, it revealed interesting details concerning comparative mouthpart and front leg morphology including quantitative shape differences of mandibles. For the first time, the predatory behaviour of several species was analysed in greater detail by using highspeed videography with the observed behavioural patterns being assigned to certain overall functions in the context of predation. By integrating the various aspects of morphology, behaviour, and performance, we obtained valuable insights into prey specialization, niche differentiation, and functional relationships between the morphology and the ecological performance of these beetles. Our study provides a foretaste of the potential data that might be obtained by a more systematic taxon sampling scheme for a comparison of the ecomorphology of certain clades (subfamilies, tribes, genera) of the “Staphylinine group” in order to attain a better understanding of the ecological drivers that have determined the evolution of this megadiverse group of beetles.

## Figures and Tables

**Table 1:** List of species considered in this study. *N*: number of individuals considered in behavioural and morphological examinations. ID numbers correspond to species data points in figures where applicable.

Species					*N* Behaviour	*N* Morphology	ID in Plots
Staphylininae	Staphylinini	Staphylinini propria	Philonthina	*Philonthus alpinus* Eppelsheim, 1875	2	2	1
				*Philonthus cruentatus* (Gmelin, 1790)	1	-	-
				*Philonthus discoideus* (Gravenhorst, 1802)	1	1	2
				*Philonthus rufipes* (Stephens, 1832)	2	1	3
				*Philonthus marginatus* (O. F. Müller, 1764)	2	2	4
				*Philonthus pseudovarians* Strand, 1941	1	-	-
				*Philonthus spinipes* Sharp, 1874	1	-	-
				*Philonthus tenuicornis* Mulsant and Rey, 1853	1	-	-
				*Philonthus umbratilis* (Gravenhorst, 1802)	1	-	-
				*Philonthus varians* (Paykull, 1789)	13	4	5
				*Bisnius sordidus* (Gravenhorst, 1802)	5	3	6
		Quediina		*Quedius (Distichalius) cinctus* (Paykull, 1790)	2	1	9
				*Quedius (Microsaurus) cruentus* (Olivier, 1795)	1	1	8
				*Quedius (Quedius) curtipennis* Bernhauer, 1908	2	1	7
	Xantholinini			*Gyrohypnus fracticornis* (O. F. Müller, 1776)	2	1	10
	Othiini			*Othius punctulatus* (Goeze 1777)	2	2	11
Paederinae	Paederini	Stilicina		*Rugilus erichsonii* (Fauvel, 1867)	1	1	12
				*Rugilus mixtus* (Lohse, 1956)	1	1	13
				*Rugilus orbiculatus* (Paykull, 1790)	1	1	14
				*Rugilus rufipes* Germar, 1836	5	4	15

**Table 2:** Squared Mahalanobis distances between tribes with *p* values of the canonical variate analysis to quantify differences between shapes. Only significant distances are shown. Abbreviations: O: Othiini; P: Philonthina; Q: Quediina; S: Stilicina; X: Xantholinini.

Tribe	D^2^	*p* Value
X vs. O	37.7	<0.0001
X vs. P	49.4	<0.05
P vs. Q	8.1	<0.05
P vs. S	128.5	<0.005
Q vs. S	98.4	<0.05

**Table 3:** Occurrence of behaviours in the various beetle species. The crosses in the table mark which rove beetle species were observed and documented performing the behaviours provided in the columns. The numbers in brackets following the species names show the total number of individuals involved.

~20 Species (47 Individuals)					Attack with Mandibles	Attack with Front Legs	Dragging the Prey	Caging the Prey with Legs	Positioning with Front Legs while Feeding	Sensation with Antennae
Staphylininae	Staphylinini	Staphylinini propria	Philonthina	*Philonthus alpinus (2)*	x		x	x	x	x
				*Philonthus cruentatus (1)*	x		x	x	x	x
				*Philonthus discoideus (1)*	x			x	x	x
				*Philonthus rufipes + P. cf rufipes (2)*	x		x	x	x	x
				*Philonthus marginatus (2)*	x	x	x	x	x	x
				*Philonthus pseudovarians (1)*	x		x		x	x
				*Philonthus spinipes (1)*	x			x	x	x
				*Philonthus tenuicornis (1)*	x		x		x	x
				*Philonthus umbratilis (1)*	x		x	x	x	x
				*Philonthus varians (13)*	x	x	x	x	x	x
				*Bisnius sordidus (5)*	x		x	x	x	x
		Quediina		*Quedius curtipennis + Q. cf curtipennis (2)*	x	x	x	x	x	x
				*Quedius (Microsaurus) cruentus (1)*	x		x	x	x	x
				*Quedius (Distalichius) cinctus (2)*	x		x		x	x
	Xantholinini			*Gyrohypnus fracticornis* (2)	x		x	x	x	x
	Othiini			*Othius punctulatus* (2)	x		x		x	
Paederinae	Paederini	Stilicina		*Rugilus erichsonii (1)*	x		x		x	x
				*Rugilus mixtus (1)*	x		x	x	x	x
				*Rugilus orbiculatus (1)*	x			x	x	x
				*Rugilus rufipes (5)*	x			x	x	x

**Table 4:** Spearman correlation matrix of variables analysed with phylogenetic independent contrast. Only variables with significant correlations are shown (significance levels: ** *p* < 0.01, * *p* < 0.05, n.s. not significant), sample size N = 14.

	Relative Number of Mites Killed	Relative Number of Collembola Killed	Relative Number of *Drosophila* Killed
**In-lever/Forebody length**	0.32 n.s.	−0.52 n.s.	0.69 ** (Figure 23B)
**Pronotum length**	0.46 n.s.	−0.60 * (Figure 23D)	0.56 * (Figure 23C)
**PC1**	0.56 * (Figure 23A)	−0.37 n.s.	0.09 n.s.

## Data Availability

Data provided in Appendix A (Table A1).

## Appendix A

**Table A1:** Table of arithmetic means obtained for morphological ratios and prey-capture ratios for each species with sample size N and standard deviation (SD). NA: calculation not possible because of small sample size.

Species	In-Lever/Mandible Length	SD	*N*	In-Lever/Out-Lever	SD	*N*	Out-Lever/Mandible Length	SD	*N*	Mandible Length/Width	SD	*N*
*P. alpinus*	0.27	0.0	2	3.62	3.3	2	0.13	0.1	2	2.91	1.1	2
*P. discoideus*	0.21	NA	1	3.95	NA	1	0.054	NA	1	3.01	NA	1
*P. rufipes*	0.22	NA	1	3.69	NA	1	0.060	NA	1	4.26	NA	1
*P. marginatus*	0.16	0.0	2	1.69	0.5	2	0.099	0.02	2	4.42	0.5	2
*P. varians*	0.26	0.0	4	4.33	2.4	4	0.070	0.03	4	3.12	0.3	4
*B. sordidus*	0.28	0.0	3	4.02	1.0	3	0.073	0.02	3	3.32	0.3	3
*Q. curtipennis*	0.27	NA	1	3.86	NA	1	0.069	NA	1	4.40	NA	1
*Q. cruentus*	0.22	NA	1	3.39	NA	1	0.065	NA	1	3.55	NA	1
*Q. cinctus*	0.28	NA	1	3.77	NA	1	0.073	NA	1	3.66	NA	1
*G. fracticornis*	0.32	NA	1	3.07	NA	1	0.11	NA	1	2.50	NA	1
*O. punctulatus*	0.34	0.0	2	4.07	0.3	2	0.083	0.00	2	2.38	NA	1
*R. erichsonii*	0.17	NA	1	2.78	NA	1	0.062	NA	1	4.27	NA	1
*R. mixtus*	0.09	NA	1	2.35	NA	1	0.040	NA	1	6.52	NA	1
*R. orbiculatus*	0.18	NA	1	5.06	NA	1	0.036	NA	1	5.40	NA	1
*R. rufipes*	0.13	0.0	4	2.93	0.6	4	0.045	0.01	4	5.28	0.4	3
**Species**	**Incisor/Mandible Length**	**SD**	***N***	**Mandible/Forebody Length**	**SD**	***N***	**In-Lever/Forebody Length**	**SD**	***N***	**Out-Lever/Forebody Length**	**SD**	***N***
*P. alpinus*	0.46	0.0	2	0.13	0.0	2	0.035	0.01	2	0.014	0.01	2
*P. discoideus*	0.30	NA	1	0.17	NA	1	0.037	NA	1	0.009	NA	1
*P. rufipes*	0.43	NA	1	0.17	NA	1	0.037	NA	1	0.010	NA	1
*P. marginatus*	0.48	0.0	2	0.15	0.0	2	0.024	0.00	2	0.015	0.00	2
*P. varians*	0.40	0.1	4	0.15	0.0	4	0.039	0.01	4	0.010	0.00	4
*B. sordidus*	0.32	0.1	3	0.19	0.0	3	0.055	0.01	3	0.014	0.00	3
*Q. curtipennis*	0.39	NA	1	0.20	NA	1	0.052	NA	1	0.014	NA	1
*Q. cruentus*	0.38	NA	1	0.17	NA	1	0.039	NA	1	0.011	NA	1
*Q. cinctus*	0.43	NA	1	0.22	NA	1	0.062	NA	1	0.016	NA	1
*G. fracticornis*	0.33	NA	1	0.14	NA	1	0.046	NA	1	0.015	NA	1
*O. punctulatus*	0.32	0.1	2	0.20	0.0	2	0.067	0.01	2	0.016	0.00	2
*R. erichsonii*	0.44	NA	1	0.18	NA	1	0.031	NA	1	0.011	NA	1
*R. mixtus*	0.45	NA	1	0.17	NA	1	0.016	NA	1	0.007	NA	1
*R. orbiculatus*	0.39	NA	1	0.19	NA	1	0.035	NA	1	0.007	NA	1
*R. rufipes*	0.50	0.0	4	0.20	0.0	4	0.026	0.00	4	0.009	0.00	4
**Species**	**Head Length/Width**	**SD**	***N***	**Head Width/Pronotum Length**	**SD**	***N***	**Relative Number of Mites Killed**	**SD**	***N***	**Relative Number of Collembola Killed**	**SD**	***N***
*P. alpinus*	1.37	0.15	2	0.68	0.0	2	0.38	NA	2	0.50	NA	2
*P. discoideus*	1.08	NA	1	0.89	NA	1	0.00	NA	1	1.00	NA	1
*P. rufipes*	1.27	NA	1	0.72	NA	1	0.33	NA	1	0.33	NA	1
*P. marginatus*	1.25	0.09	2	0.75	0.1	2	0.26	NA	2	0.47	NA	2
*P. varians*	1.33	0.07	4	0.69	0.1	4	0.25	NA	13	0.27	NA	13
*B. sordidus*	0.87	0.75	3	0.80	0.1	3	0.44	NA	5	0.24	NA	5
*Q. curtipennis*	1.22	NA	1	0.85	NA	1	0.25	NA	1	0.25	NA	1
*Q. cruentus*	1.23	NA	1	0.88	NA	1	0.30	NA	1	0.40	NA	1
*Q. cinctus*	1.22	NA	1	0.91	NA	1	0.00	NA	2	0.50	NA	2
*G. fracticornis*	1.54	NA	1	0.73	NA	1	0.14	NA	1	0.71	NA	1
*O. punctulatus*	1.36	0.17	2	0.75	0.0	2	0.33	NA	2	0.17	NA	2
*R. erichsonii*	1.22	NA	1	1.19	NA	1	0.17	NA	1	0.83	NA	1
*R. mixtus*	1.13	NA	1	1.18	NA	1	0.00	NA	1	1.00	NA	1
*R. orbiculatus*	1.28	NA	1	1.07	NA	1	0.00	NA	1	1.00	NA	1
*R. rufipes*	1.14	0.09	4	1.12	0.0	4	0.00	NA	5	0.93	NA	5
**Species**	**Relative Number of *Drosophila* Killed**	**SD**	***N***									
*P. alpinus*	0.13	NA	2									
*P. discoideus*	0.00	NA	1									
*P. rufipes*	0.33	NA	1									
*P. marginatus*	0.26	NA	2									
*P. varians*	0.48	NA	13									
*B. sordidus*	0.32	NA	5									
*Q, curtipennis*	0.50	NA	1									
*Q. cruentus*	0.30	NA	1									
*Q. cinctus*	0.50	NA	2									
*G. fracticornis*	0.14	NA	1									
*O. punctulatus*	0.50	NA	2									
*R. erichsonii*	0.00	NA	1									
*R. mixtus*	0.00	NA	1									
*R. orbiculatus*	0.00	NA	1									
*R. rufipes*	0.07	NA	5									
